# Seasonality and depth distribution of the abundance and activity of ammonia oxidizing microorganisms in marine coastal sediments (North Sea)

**DOI:** 10.3389/fmicb.2014.00472

**Published:** 2014-09-05

**Authors:** Yvonne A. Lipsewers, Nicole J. Bale, Ellen C. Hopmans, Stefan Schouten, Jaap S. Sinninghe Damsté, Laura Villanueva

**Affiliations:** Department of Marine Organic Biogeochemistry, NIOZ Royal Netherlands Institute for Sea ResearchDen Burg, Netherlands

**Keywords:** Thaumarchaeota, anammox bacteria, ammonia oxidizing bacteria (AOB), ammonia oxidizing Archaea (AOA), *amo*A gene, *hzs*A gene, intact polar lipids (IPLs)

## Abstract

Microbial processes such as nitrification and anaerobic ammonium oxidation (anammox) are important for nitrogen cycling in marine sediments. Seasonal variations of archaeal and bacterial ammonia oxidizers (AOA and AOB) and anammox bacteria, as well as the environmental factors affecting these groups, are not well studied. We have examined the seasonal and depth distribution of the abundance and potential activity of these microbial groups in coastal marine sediments of the southern North Sea. This was achieved by quantifying specific intact polar lipids as well as the abundance and gene expression of their 16S rRNA gene, the ammonia monooxygenase subunit A (*amo*A) gene of AOA and AOB, and the hydrazine synthase (*hzs*A) gene of anammox bacteria. AOA, AOB, and anammox bacteria were detected and transcriptionally active down to 12 cm sediment depth. In all seasons, the abundance of AOA was higher compared to the AOB abundance suggesting that AOA play a more dominant role in aerobic ammonia oxidation in these sediments. Anammox bacteria were abundant and active even in oxygenated and bioturbated parts of the sediment. The abundance of AOA and AOB was relatively stable with depth and over the seasonal cycle, while anammox bacteria abundance and transcriptional activity were highest in August. North Sea sediments thus seem to provide a common, stable, ecological niche for AOA, AOB, and anammox bacteria.

## INTRODUCTION

Nitrogen is an essential component for living organisms and thus plays a critical role in controlling primary production (Gruber and Galloway, 2008). Microbial aerobic and anaerobic nitrogen cycling processes such as nitrification, denitrification and anaerobic ammonium oxidation are tightly coupled in marine sediments where 50% of marine nitrogen removal occurs (Zhang et al., 2013, and references therein). The discovery of ammonia-oxidizing archaea (AOA; Könneke et al., 2005; Wuchter et al., 2006) belonging to the phylum Thaumarchaeota (Brochier-Armanet et al., 2008), and bacteria of the Planctomycetes phylum performing the anaerobic ammonia oxidation (anammox) reaction (Strous et al., 1999; Kuypers et al., 2003), has changed our perspective on the marine nitrogen cycle and the involved microbial key-players over the last decade. Ammonia oxidation is the first and rate-limiting step of nitrification in which ammonia is oxidized to nitrite by AOA and ammonia oxidizing bacteria (AOB; see Prosser and Nicol, 2008 for a review). The anammox process involves the combination of ammonium with nitrite to form dinitrogen gas which is then released from the system (Kuenen, 2008).

Several studies focusing on ammonia oxidation in marine sediments have suggested that AOA have a more prominent role in marine nitrification than AOB based on the higher abundance of AOA *amo*A gene copies (Beman and Francis, 2006; Mosier and Francis, 2008; Santoro et al., 2008; Abell et al., 2010). Proposed environmental controls shaping the distribution and activity of AOA in marine and estuarine surface sediments include the presence of sulfide, salinity, dissolved oxygen (DO) concentration, the availability of phosphorous, temperature, and primary production (Mosier and Francis, 2008; Sahan and Muyzer, 2008; Erguder et al., 2009; Bernhard et al., 2010; Dang et al., 2010; Sakami, 2012; Bale et al., 2013). Beman et al. (2012) studied the seasonal distribution of AOA and AOB *amo*A gene abundance in Catalina Harbor (CA, USA) down to 10 cm sediment depth and showed higher abundances of AOA and AOB *amo*A genes during summer compared to winter months as well as coinciding higher ^15^NH_4_^+^ oxidation rates. For anammox bacteria, studies have shown that temperature, organic carbon content, nitrite concentration, water depth, sediment characteristics, and bioturbation play a role in their distribution, abundance, and activity in marine and estuarine sediments (Dalsgaard and Thamdrup, 2002; Engström et al., 2005; Meyer et al., 2005; Dale et al., 2009; Jaeschke et al., 2009; Dang et al., 2010; Li et al., 2010; Neubacher et al., 2011, 2013; Laverock et al., 2013; Zhang et al., 2013). However, most of the studies have focused on the spatial distribution of anammox bacteria abundance and activity only in surface sediments (Dalsgaard and Thamdrup, 2002; Thamdrup and Dalsgaard, 2002; Engström et al., 2005; Nicholls and Trimmer, 2009; Bale et al., 2014). Limited studies have been focused on the abundance of anammox bacteria with sediment depth or the impact of seasonality on this group (Jaeschke et al., 2009; Zhang et al., 2013). Anammox bacteria abundance and activity has been previously detected using anammox specific ladderane biomarker lipids in combination with ^15^N-labeling experiments in continental shelf and slope sediments of the Irish and Celtic Sea down to eight centimeters depth with spatial differences (Jaeschke et al., 2009). Recently, anammox bacteria abundance was shown to be higher during summer compared to the winter months in surface sediments of three hyper-nutrified estuarine tidal flats of Laizhou Bay (Bohai Sea, China) through the quantification of anammox bacteria 16S rRNA gene (Zhang et al., 2013). A study by Laverock et al. (2013) indicated that the temporal variation in the abundance of N-cycling functional genes is directly influenced by bioturbation activity varying between different bacterial and archaeal N-cycling genes. The relative abundance of AOB *amo*A genes is significantly affected by bioturbation whereas the relative abundance of AOA *amo*A seems to be controlled by other factors (Laverock et al., 2013). Interestingly, bioturbation and mixing can extend the area of nitrate reduction in sediments leading to the production of nitrite which would fuel the anammox process (Meyer et al., 2005).

The aim of our study was to determine differences in abundance and activity of ammonia oxidizers with sediment depth (down to 12 cm depth) in marine sediments from the southern North Sea, a continental shelf sea. To address this issue, we quantified the abundance of 16S rRNA gene of ammonia oxidizer groups (AOA, AOB, and anammox bacteria), metabolic genes (*amo*A of AOA and AOB, and hzsA gene of anammox bacteria), and the intact polar membrane lipids specific for anammox bacteria (i.e., ladderane lipids, Sinninghe Damsté et al., 2002b), and Thaumarchaeota (i.e., crenarchaeol; Sinninghe Damsté et al., 2002a). The potential transcriptional activity of the target genes was analyzed to elucidate the potential activity of the target organisms in the sediment. Our results suggest that both aerobic and anaerobic ammonia oxidizers coexist in the same niche and are actively involved in the nitrogen cycle in oxygenated and bioturbated sediments in the North Sea.

## MATERIALS AND METHODS

### STUDY SITE AND SAMPLING

The Oyster ground, an almost circular depression with a maximum depth of 50 m located in the southern North Sea (Weston et al., 2008), is a temporary deposition center for sediment (Raaphorst et al., 1998), which plays an important role in the carbon and nitrogen cycle in the region (Weston et al., 2008). The sedimentation of organic matter in this region leads to organic rich and muddy sediment, which supports high levels of benthic fauna (Duineveld et al., 1991; van Raaphorst et al., 1992).

Sediment cores were collected at a station (4°33.01′ E, 54°13.00′ N) in the Oyster ground during three cruises on board of the *R/V* Pelagia in February, May, and August 2011. Sediment was collected with 10 cm diameter multicores. Bottom water of the overlaying water was collected using a syringe and filtered (through a 0.45 μm 25 mm Acrodisc HT Tuffryn Membrane syringe filter) for nutrient analysis. The cores were sliced into 1 cm slices using a hydraulic slicer or a manual slicer. Samples were collected for lipid and DNA/RNA analysis and kept at -80°C (DNA/RNA) and -40°C (lipids) until processing.

Bottom water temperature, water depth and salinity were measured using a SBE911+ conductivity-temperature depth (CTD) system (Seabird Electronics, Inc., WA, USA). The oxygen concentration in the water column was measured using a DO sensor SBE 43 (Seabird Electronics, Inc., WA, USA) integrated in the CTD system.

### PHYSICOCHEMICAL PARAMETERS

Pore water was extracted from 2.5 mm sediment slices from 0 to 2 cm depth, and from 1 cm sediment slices from 2 to 12 cm depth by centrifugation (∼4000 × *g*, 5 min, through a 0.45 μm 25 mm Acrodisc HT Tuffryn Membrane syringe filter), and the obtained concentrations of the first 2 cm were averaged for the first and second centimeter of the sediment to estimate pore water nutrient concentrations in a 1 cm resolution. Pore water samples were stored in pre-rinsed pony vials and NH_4_^+^, NO_3_^-^, NO_2_^-^, and PO_4_^3-^ concentrations were analyzed as described by Bale et al. (2013). The total organic carbon (TOC) and total organic nitrogen (TON) content per gram of freeze dried sediment were determined after acidification of freeze dried sediment (0.5-1 g) with 2 N HCl. Residues were analyzed by using a Thermo Finnigan Delta plus isotope ratio monitoring mass spectrometer (irmMS) connected to a Flash 2000 elemental analyzer (Thermo Fisher Scientific, Milan, Italy).

### INTACT POLAR LIPID EXTRACTION AND ANALYSIS

Intact polar lipids (IPLs) were extracted following Bale et al. (2013). Hexose-phosphohexose (HPH) crenarchaeol, a specific biomarker lipid for AOA (Schouten et al., 2008), was analyzed using high performance liquid chromatography mass spectrometry (HPLC/MS) as described by Pitcher et al. (2011a). Due to the lack of a standard the results are reported here as response unit per gram of sediment (r.u. g^-1^). The anammox bacteria specific intact ladderane phospholipid, C_20_-[3]-monoether ladderane, attached to a phosphatidylcholine (PC) headgroup (PC-monoether ladderane) was analyzed by HPLC/MS following Jaeschke et al. (2009) and quantified using an external standard consisting of isolated PC-monoether ladderane.

### DNA/RNA EXTRACTION

DNA and RNA from sediment cores were extracted by using the DNA and RNA PowerSoil®; Total Isolation Kit, respectively (Mo Bio Laboratories, Inc., Carlsbad, CA, USA). Nucleic acid concentrations were quantified spectrophotometrically (Nanodrop, Thermo Scientific, Wilmington, DE, USA) and checked by agarose gel electrophoresis for integrity. Extracts were kept frozen at -80°C. The RNA extracts were treated with RNase-free DNase (DNA-*free*^TM^, Ambion Inc., Austin, TX, USA). RNA quality and concentration were estimated by the Experion RNA StdSens Analysis Kit (Bio-Rad Laboratories, Hercules, CA, USA). DNA contamination was checked by PCR using RNA as a template.

### REVERSE TRANSCRIPTION (RT)-PCR

Reverse transcription (RT) was performed with the Enhanced Avian First Strand synthesis kit (Sigma–Aldrich Co., St Louis, MO, USA) using random nonamers as described previously (Holmes et al., 2004). Two negative controls lacking reverse transcriptase or RNA were included. PCR reactions were performed as described above to confirm the transcription to complementary DNA (cDNA) and the negative controls using the RT reaction as a template.

### QUANTITATIVE PCR (qPCR) ANALYSIS

Quantitative PCR (qPCR) analyses were performed on a Biorad CFX96^TM^ Real-Time System/C1000 Thermal cycler equipped with CFX Manager^TM^ Software. Gene copy numbers of the Thaumarchaeota group 1.1a were estimated by using the 16S rRNA gene primers MCGI-391F/MCGI-554R (Coolen et al., 2007). The AOA *amo*A gene was amplified using the primer combination AmoA-ModF/AmoA-ModR (Yakimov et al., 2011). Abundance of AOB 16S rRNA gene was estimated using primers CTO189F/CTO654R as described by Kowalchuk et al. (1997). Gene copy numbers of the AOB *amo*A gene were estimated using the primer set AOB-*amo*AF/AOB-*amo*AR new (Rotthauwe et al., 1997; Hornek et al., 2006). Abundance of the anammox bacteria 16S rRNA gene was estimated using primers Brod541F/Amx820R as described by Li et al. (2010). Additionally, the anammox bacteria *hzs*A gene was quantified using the primer combination *hzs*A_1597F/*hzs*A_1857R as described by Harhangi et al. (2012; see Table S1 in Supplementary Materials for details). All qPCR reactions were performed in triplicate with standard curves from 10^0^ to 10^7^ molecules per microliter. Standard curves were generated as described before (Pitcher et al., 2011b). Gene copies were determined in triplicates on diluted DNA extracts (1:10) and on cDNA extracts. The reaction mixture (25 μL) contained 1 U of PicoMaxx high fidelity DNA polymerase (Stratagene, Agilent Technologies, Santa Clara, CA, USA) 2.5 μL of 10× PicoMaxx PCR buffer, 2.5 μL 2.5 mM of each dNTP, 0.5 μL BSA (20 mg mL^-1^), 0.02 pmol μL^-1^ of primers, 10,000 times diluted SYBR Green®; (Life Technologies, Carlsbad, CA, USA; optimized concentration), 0.5 μL 50 mM of MgCl_2_, and ultra-pure sterile water. All reactions were performed in iCycler iQ^TM^ 96-well plates with optical tape (Bio-Rad, Hercules, CA, USA). Specificity of the reaction was tested with a gradient melting temperature assay. The cycling conditions for the qPCR reaction were the following: 95°C, 4 min; 40–45× [95°C, 30 s; melting temperature (T_m_), 40 s; 72°C, 30 s]; final extension 80°C, 25 s. Specificity for qPCR reaction was tested on agarose gel electrophoresis and with a melting curve analysis (50–95°C; with a read every 0.5°C held for 1 s between each read) in order to identify unspecific PCR products such as primer dimers or fragments with unexpected fragment lengths. Melting temperature, PCR efficiencies (*E*), and correlation coefficients for standard curves are listed in Table S2 in Supplementary Materials.

### PCR AMPLIFICATION AND CLONING

Amplifications of the anammox bacteria 16S rRNA gene, AOA, and AOB *amo*A genes were performed with the primer pairs specified above (Table S1 in Supplementary Materials). PCR reaction mixture was the following (final concentration): Q-solution (PCR additive, Qiagen, Valencia, CA, USA) 1×; PCR buffer 1×; BSA (200 μg mL^-1^); dNTPs (20 μM); primers (0.2 pmol μL^-1^); MgCl_2_ (1.5 mM); 1.25 U Taq polymerase (Qiagen, Valencia, CA, USA). PCR conditions for these amplifications were the following: 95°C, 5 min; 35× [95°C, 1 min; T_m_, 1 min; 72°C, 1 min]; final extension 72°C, 5 min. PCR products were gel purified (QIAquick gel purification kit, Qiagen, Valencia, CA, USA), cloned in the TOPO-TA cloning®; kit (Life Technologies, Carlsbad, CA, USA), and transformed in *E. coli* TOP10 cells following the manufacturer’s recommendations. Recombinant plasmidic DNA was sequenced using M13R (5′-CAG GAA ACA GCT ATG AC-3′) primer by Macrogen Inc. (Amsterdam, Netherlands).

### PHYLOGENETIC ANALYSIS

Sequences were analyzed for the presence of chimeras using the Bellerophon tool at the GreenGenes website (http://greengenes.lbl.gov/). Sequences were aligned with Mega5 software (Tamura et al., 2011) by using the alignment method ClustalW. Partial sequences of the archaeal *amo*A gene generated in this study were added to the *amo*A gene reference tree provided in Pester et al. (2012) using the ARB Parsimony tool (Ludwig et al., 2004). Phylogenetic trees of AOB *amo*A and the anammox bacterial 16S rRNA genes were computed with the Neighbor-Joining method (Saitou and Nei, 1987) in the Mega5 software. The evolutionary distances were estimated using the Jukes-Cantor method (Jukes and Cantor, 1969) with a bootstrap test of 1000 replicates. Sequences were deposited in NCBI with the following accession numbers: KJ807530–KJ807556 for partial sequences of the archaeal *amo*A gene, KJ807557–KJ807597 for partial bacterial *amo*A gene sequences and KJ807598–KJ807609 for partial anammox bacterial 16S rRNA gene sequences.

### STATISTICAL ANALYSIS

Spearman’s rank order correlation coefficient (*r*_s_) analysis was performed using the SigmaPlot^TM^ (12.0) Exact Graphs and Data Analysis (Systat Inc., San Jose, CA, USA). Additional multivariate analysis did not show additional correlations of environmental data with gene and expression data therefore results were not included in this study.

## RESULTS

### PHYSICOCHEMICAL CONDITIONS

Oxygen concentrations of the water column corresponding to the sampling time and location of sediment core sampling are shown in Figure S1 in Supplementary Materials. In February, oxygen concentrations decreased throughout the water column from 301.3 to 299 μM (300 μM on average). In May, the oxygen concentration was slightly lower compared to February (282 μM on average) and decreased between 17.8 and 22.8 m water depth from about 287 to 278 μM. Lowest oxygen concentrations were detected in August compared to February and May (230 μM on average) and decreased between 17.8 and 24.8 m water depth from 242 to 217.25 μM.

Physicochemical parameters of the bottom water during the cruises of February (winter), May (spring), and August (summer) are shown in Table S3 in Supplementary Materials. Bottom water temperatures ranged from 5 to 15.4°C. Salinity values were stable throughout the year ranging from 34.3 to 34.7 practical salinity units (psu). Ammonia (NH_4_^+^) concentrations in the bottom water ranged between 1.6 and 3 μM with a maximum concentration in February. Bottom water nitrite (NO_2_^-^) concentrations varied from 0.6 μM in February to 0.1 μM in May and August. Highest nitrate (NO_3_^-^) concentrations were detected in February (1 μM) and lowest in August (0.5 μM).

**FIGURE 1:**
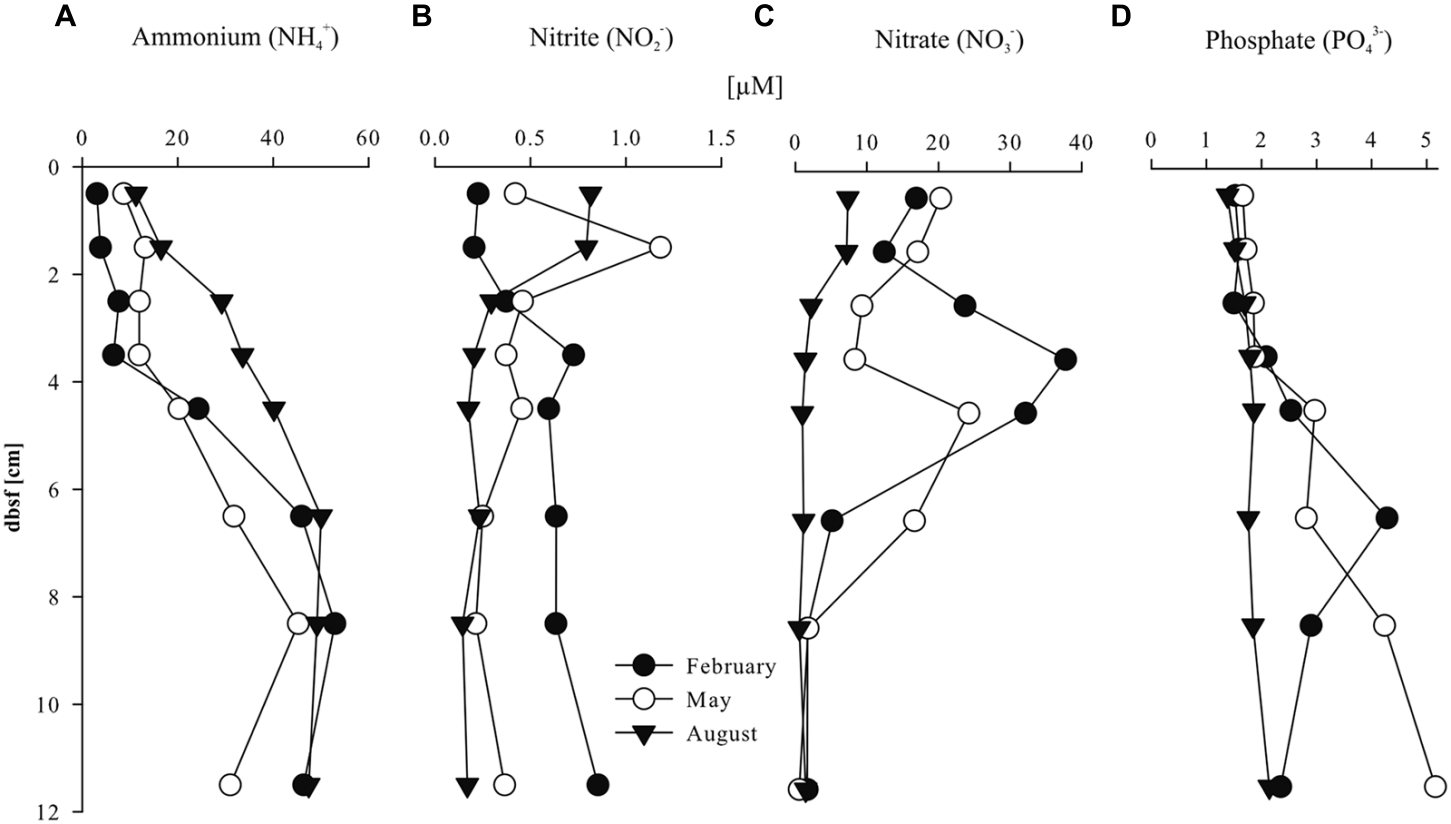
**Sediment pore water nutrients: (A) NH_4_^+^; (B) NO_2_^-^; (C) NO_3_^-^ and (D) PO_4_^3+^ (μM) with sediment depth (cm) below sea floor (bsf)**.

Pore water concentrations of NH_4_^+^ ranged between 3 and 53 μM in all three seasons (**Figure 1**). The depth trend of ammonia concentrations in the pore water was similar in all seasons with lower concentrations in the upper 4 cm below sea floor (bsf; 3–34 μM) compared to increasing concentrations of the underlying layers (20–53 μM) down to 12 cm bsf. Highest NH_4_^+^ concentrations were detected in August (40–53 μM) compared to February and May. Nitrite (NO_2_^-^) concentrations ranged between 0.1 and 1.2 μM in all seasons (**Figure 1**). Depth profiles of pore water nitrite concentrations showed different seasonal trends. In February, nitrite concentrations were relatively low (0.3 μM) in the first 3 cm bsf and increased with depth (0.6 μM) whereas in May and August, nitrite concentrations were higher between 1 and 2 cm bsf (0.8 μM) compared to the underlying sediment layers (0.4 and 0.2 μM, respectively). Pore water nitrate (NO_3_^-^) concentrations strongly varied with sediment depth and season (**Figure 1**). In February and May, nitrate concentrations were highly variable with depth and reached maximum values between 3 and 4 cm (38 μM) and 4 and 5 cm bsf (24 μM), while the concentration strongly decreased in the underlying layers. In August, lower nitrate concentrations were detected compared to the other seasons with slightly elevated values in the first 2 cm bsf (7.3 μM) and lower concentrations in the underlying layers (1.3 μM). Phosphate concentrations ranged between 1.4 and 5.2 μM in all seasons (**Figure 1**). Pore water phosphate concentrations were stable in the first 4 cm bsf (1.7 μM, averaged) in all three seasons. In February and May, phosphate concentrations increased slightly (3.4 μM, averaged) in the underlying sediment layers whereas the depth profile remained stable in August. TOC and TON percentages of the sediment were relatively stable in all seasons (between 0.15 and 0.38% and 0.02 and 0.08%, respectively), with highest values in May between 1 and 2 cm and 4 and 5 cm bsf (Table S4 in Supplementary Materials).

### ABUNDANCE, DISTRIBUTION, ACTIVITY, AND DIVERSITY OF AOA AND AOB

**FIGURE 2:**
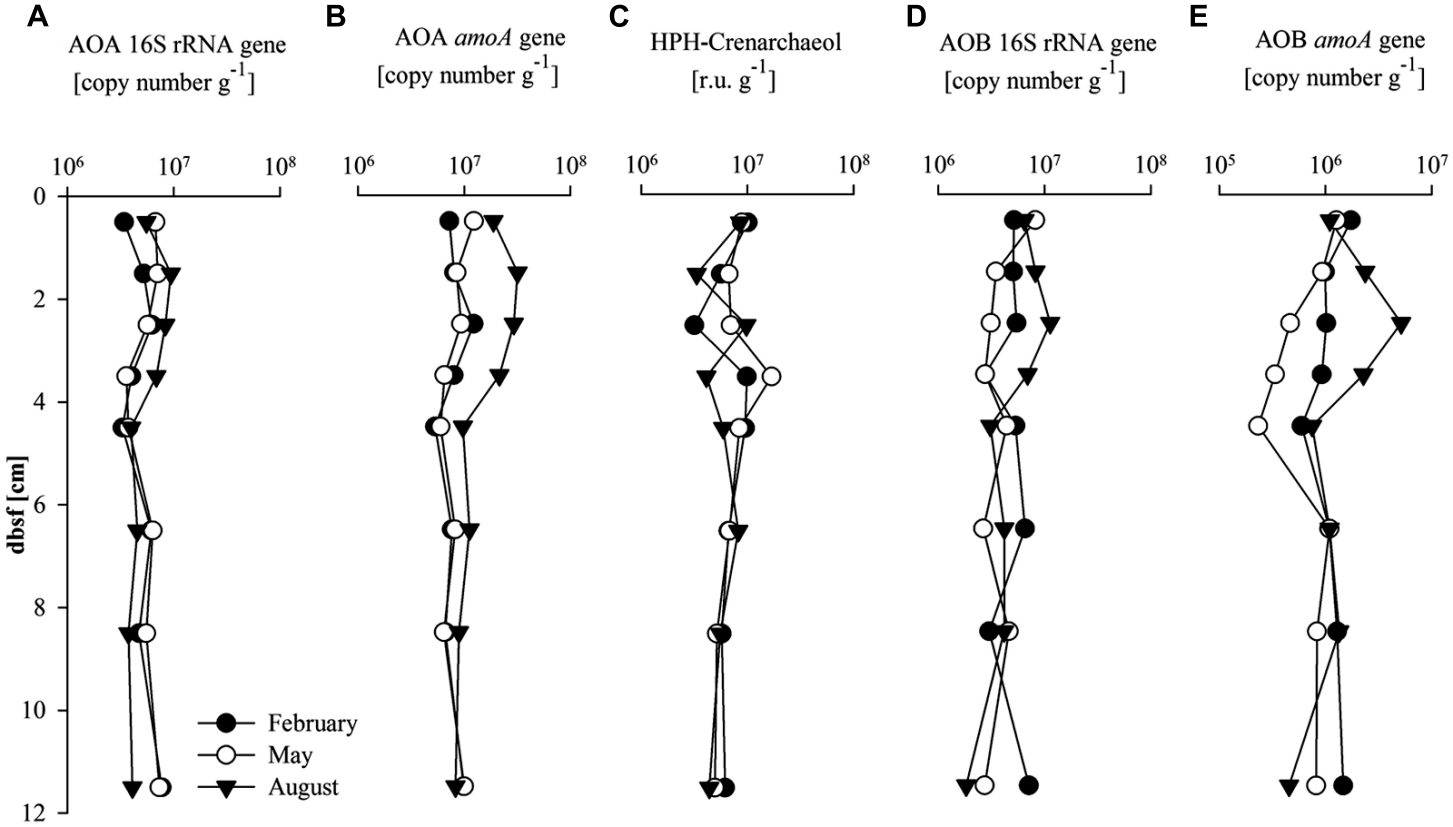
**Abundance of (A) ammonia oxidizing Archaea (AOA) 16S rRNA gene (copy number g^-1^); (B) AOA *amo*A gene (copy number g^-1^); (C) Hexose phosphohexose (HPH)-crenarchaeol (response units g^-1^), and (D) AOB 16S rRNA gene (copy number g^-1^); (E) AOB *amo*A gene (copy number g^-1^) with sediment depth (cm) below sea floor (bsf)**.

The abundance of AOA in the sediments was determined by quantification of the 16S rRNA and *amo*A gene copies, as well as by quantification of HPH-crenarchaeol (**Figures 2A–C**). The seasonal variations in depth profiles of AOA 16S rRNA and *amo*A gene abundance were generally similar (**Figures 2A,B**). Both AOA gene abundances were higher between 0 and 5 cm (6 × 10^6^ and 1.4 × 10^7^ gene copies g^-1^, respectively) compared to 5 and 12 cm bsf (5 × 10^6^ and 8.2 × 10^6^ gene copies g^-1^, respectively). Gene copy numbers of the AOA *amoA* gene were higher in August in comparison to the values observed in February and May (1.4-fold and 2.7-fold higher, respectively) for the 0–5 cm interval. HPH-crenarchaeol concentration was variable over the first 5 cm bsf (between 3 × 10^6^ and 17 × 10^6^ r.u. g^-1^), but stable values (around 5.9 × 10^6^ r.u. g^-1^) were detected in the layers underneath. No clear seasonal differences were detected (**Figure 2C**).

The abundance of AOB was determined by quantifying the AOB 16S rRNA and *amo*A gene (**Figures 2D,E**). Values were relatively stable with depth with seasonal variations between 0 and 4 cm bsf. Abundance of the AOB 16S rRNA gene showed values similar to the AOA 16S rRNA gene abundance in February and August (on average 5.1 × 10^6^ and 5.8 × 10^6^ gene copies g^-1^, respectively), while in May AOB 16S rRNA gene copy numbers were lower on average compared to the AOA 16S rRNA gene copy number (4.0 × 10^6^ gene copies g^-1^, 1.4-fold lower). The AOB *amo*A gene abundance followed the same seasonal trends as that of the AOB 16S rRNA gene, AOA 16S rRNA and *amo*A gene abundances, with higher values in August, but absolute values were one order of magnitude lower than AOA *amo*A gene copy numbers (2.3 × 10^5^–5.2 × 10^6^ gene copies g^-1^). AOB *amo*A gene abundance was highest in the 0–5 cm depth interval except in May when values between 2–5 cm and in the 5–12 cm depth interval were lower than in the surface sediment.

**FIGURE 3:**
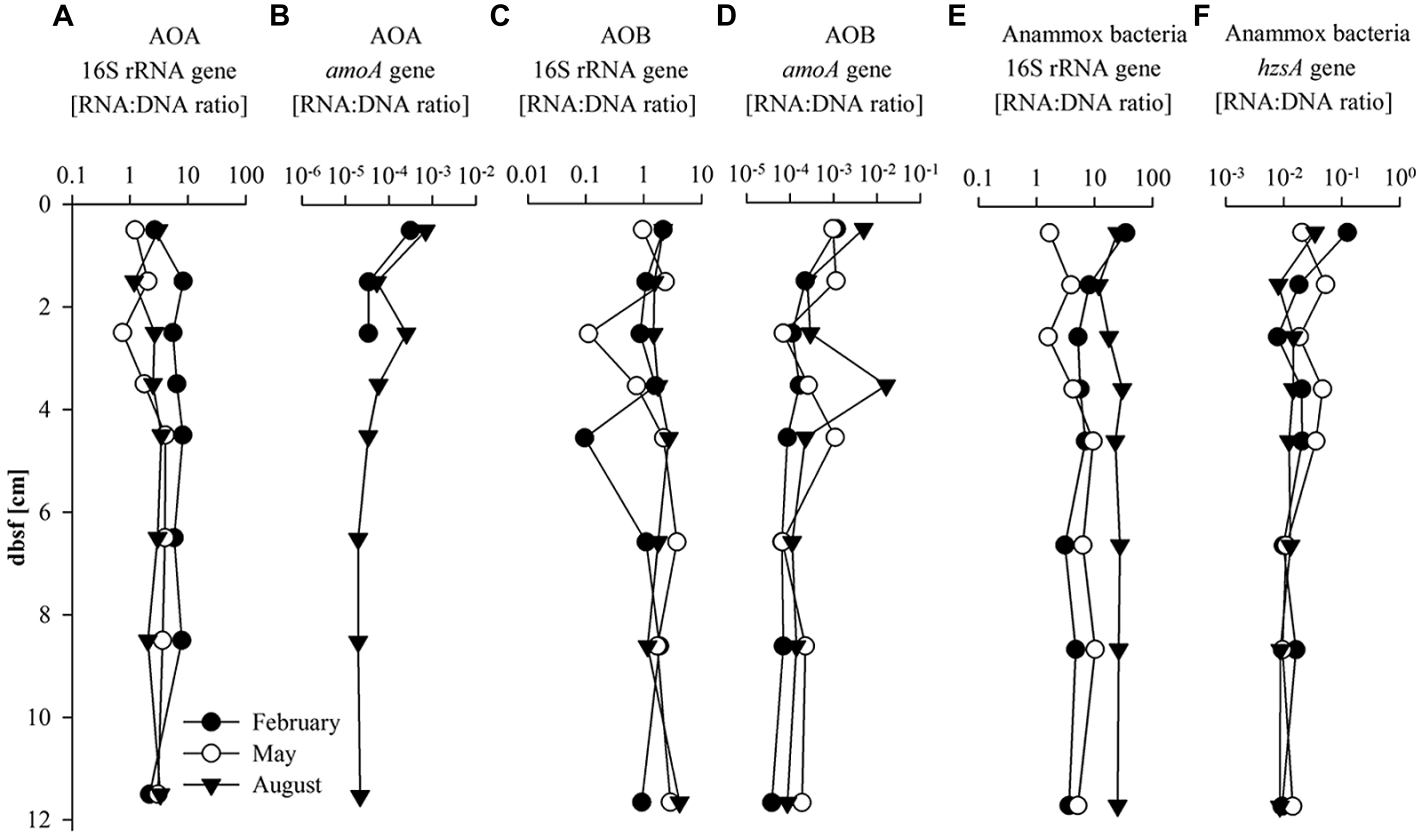
**Transcriptional activity given as RNA:DNA ratio of (A) AOA 16S rRNA gene; (B) AOA *amo*A gene; (C) AOB 16S rRNA gene, (D) AOB *amo*A; (E) Anammox 16S rRNA gene, and (F) Anammox bacteria *hzs*A gene with sediment depth (cm) below sea floor (bsf)**.

The RNA:DNA ratios of the AOA and AOB 16S rRNA and *amo*A genes were calculated as an indicator of the potential transcriptional activity of the targeted microbial group (**Figures 3A–D**). In all three seasons, AOA 16S rRNA gene RNA:DNA ratio showed relatively stable values down core ranging between 0.9 and 5.0 and 1.2 and 3.4 in May and August but reached slightly higher values (2.2–8.2) in February (**Figure 3A**). AOB 16S rRNA gene RNA:DNA ratio was between 0.1 and 2.1 with a minimum of 0.1 between 4 and 5 cm bsf in February and 0.1 and 3.7 with a minimum between 2 and 3 cm bsf in May. The AOB 16S rRNA gene RNA:DNA ratio was slightly higher (1.2–4.1) in August (**Figure 3C**). Values were relatively stable with depth in August whereas in February and May values were more variable. Compared to the RNA:DNA ratio of the AOA 16S rRNA gene, the AOB 16S rRNA gene RNA:DNA ratio was lower (fivefold in February, 1.5-fold in May and 1.3-fold lower in August) in all seasons (**Figures 3A,C**). AOA *amo*A gene transcripts (**Figure 3B**) were only detected in February in the first 3 cm and in August throughout the core, and were under the detection limit in May. AOB *amo*A gene RNA:DNA ratio (**Figure 3D**) varied between 4 × 10^-5^ and 2 × 10^-2^ in all seasons with a slight decrease with depth and highest values in August.

The diversity of AOA and AOB was evaluated in two selected depth intervals (0–1 and 9–10 cm bsf) of the sediment core recovered in August by targeting the AOA *amo*A gene and the AOB *amo*A gene, respectively (Figures S2 and S3 in Supplementary Materials). Amplified AOA *amo*A gene sequences were closely related to AOA *amo*A gene sequences previously isolated from marine and estuarine environments and did not cluster according to depth (Figure S2 in Supplementary Materials). Sequences were mainly affiliated to the *Nitrosopumilus* subclusters 12 and 16, also known as the stable marine cluster, or to the *Nitrosopumilus* subcluster 4.1, known as the estuarine cluster, according to the phylogenetic classification proposed by Pester et al. (2012). The AOB *amo*A gene sequences were affiliated to AOB *amo*A gene sequences previously isolated from estuarine sediments. Sequences were closely related to the AOB *amo*A gene sequences of *Nitrospira* sp. (accession number X90821.1) and *Nitrosolobus multiformis* (accession number AF 042171.1). No clustering of AOB *amo*A sequences according to depth was observed (Figure S3 in Supplementary Materials).

### ABUNDANCE, DISTRIBUTION, ACTIVITY, AND DIVERSITY OF ANAMMOX BACTERIA

**FIGURE 4:**
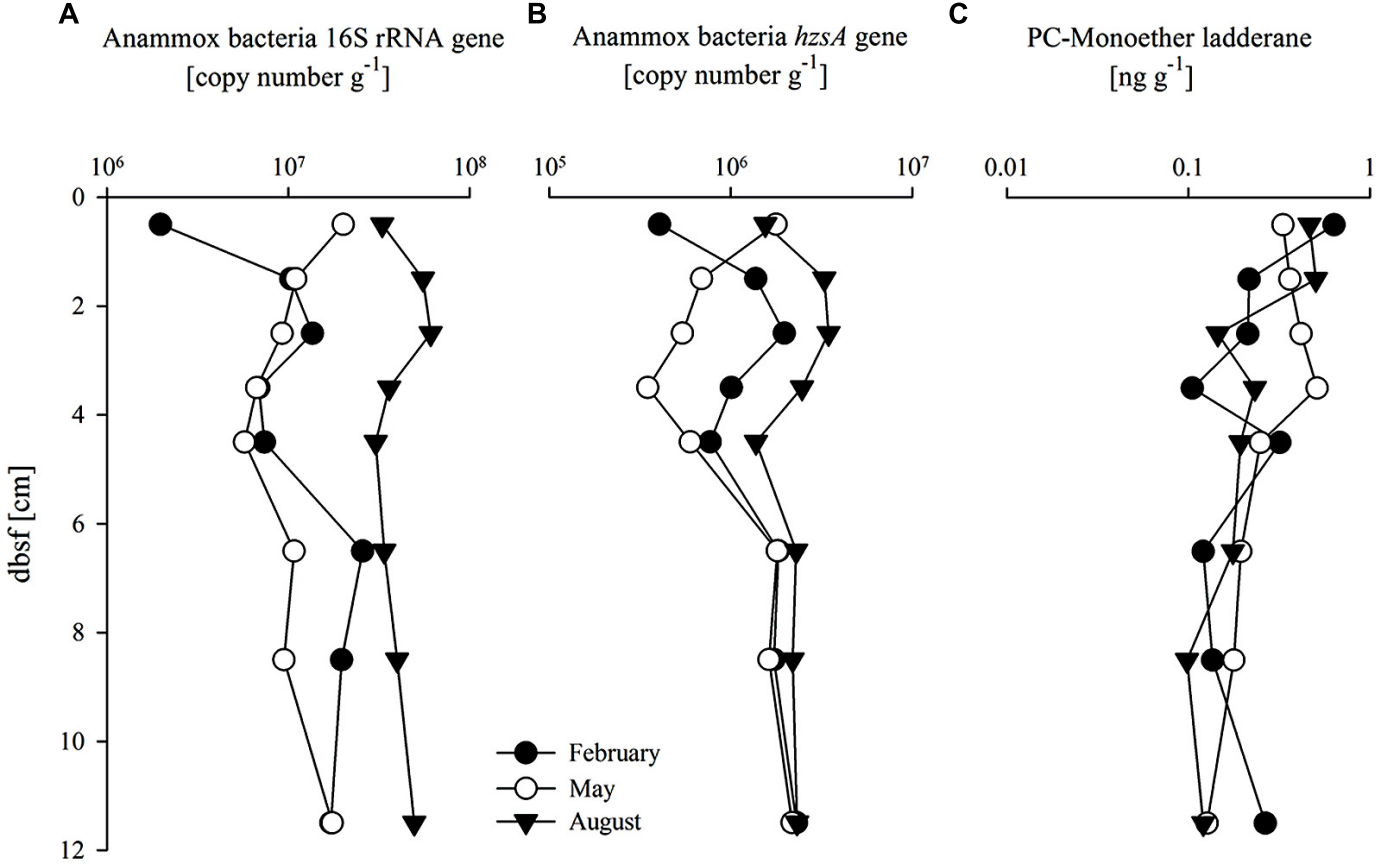
**Abundance of (A) Anammox bacteria 16S rRNA gene (copy number g^-1^); (B) Anammox bacteria *hzs*A gene (copy number g^-1^), and (C) PC-monoether ladderane (ng g^-1^) with sediment depth (cm) below sea floor (bsf)**.

The abundance and distribution of anammox bacteria were estimated by quantification of anammox bacteria 16S rRNA and *hzs*A gene copy number, as well as by quantification of the PC-monoether ladderane (**Figures 4A–C**). Depth profiles of anammox bacteria 16S rRNA and *hzs*A gene abundances followed similar trends. Gene copy numbers of the *hzs*A gene were one order of magnitude lower (between 3.5 × 10^5^ and 2 × 10^6^ copies g^-1^) than anammox bacteria 16S rRNA gene copy numbers in all three seasons (**Figures 4A,B**). Values were higher in August compared to those in February and May, especially for the anammox bacteria 16S rRNA gene (3.7-fold higher). The RNA:DNA ratio for the anammox bacteria 16S rRNA gene varied between 1.6 and 34.6 (**Figure 3E**) with higher values in August compared to February and May (on average fourfold higher). The *hzs*A gene RNA:DNA ratio (**Figure 3F**) depth profiles showed relatively stable values (between 0.008 and 0.125) throughout the core without significant seasonal differences. The PC-monoether ladderane concentration (**Figure 4C**) was variable over the first 5 cm bsf (between 0.25 and 1.25 ng g^-1^), while in the underlying sediment layers values decreased slightly to more stable values (approximately 0.36 ng g^-1^) down to 12 cm bsf. There was no clear seasonal difference observed.

The diversity of anammox bacteria was evaluated in two selected depths (0–1 and 9–10 cm bsf) of the sediment core in August by targeting the 16S rRNA gene of anammox bacteria. The sequences were closely related to “*Candidatus Scalindula* marina” (accession number EF602038) and “*Candidatus Scalindula* brodae” (accession number AY254883) and to sequences previously detected in waters of marine oxygen minimum zones (Woebken et al., 2008). Anammox bacterial 16S rRNA gene sequences from different depths were closely related to each other (Figure S4 in Supplementary Materials).

## DISCUSSION

### AEROBIC AMMONIA OXIDIZERS

In this study, we defined two niches in the Oyster ground sediment based on the differences in physicochemical parameters and in the abundance of the target organisms, i.e., the upper 4 cm of the sediment, and the underlying sediment. Ammonia concentration in the pore water of the upper part of the sediment (0–4 cm) was consistently lower than in deeper sediment layers (4–12 cm; **Figure 1**), suggesting ammonia diffusion from below formed by mineralization of organic matter by ammonification or dissimilatory nitrate reduction to ammonium (DNRA). In addition, the abundance of AOA and AOB (as well as anammox bacteria, see below) was higher and more affected by seasonality in the upper part than deeper in the sediment, which apparently is a more stable compartment. The higher variability observed in the upper part of the sediment is in agreement with the seasonal mixing of the bottom water layers and sediment deposition dynamics in this area (Greenwood et al., 2010).

Oxygen availability is expected to play an important role in the dynamics of ammonia oxidizing microorganisms in marine sediments. In the summer months, water stratification leads to a decrease in the DO concentration in the bottom water in the Oyster ground area (Greenwood et al., 2010; see Figure S1 in Supplementary Materials). Indeed, bottom water hypoxia has been shown to cause a decrease in oxygen penetration depth and oxygen consumption in Oyster ground sediments by induced short-term hypoxia in intact sediment cores (Neubacher et al., 2011). Lohse et al. (1996) reported an oxygen penetration depth (OPD) of 1.7 cm in February and 0.5 cm in August in sediment at a station close to the Oyster ground using oxygen microelectrodes, suggesting permanently anoxic conditions from a depth of approximately >2 cm throughout the year. However, the OPD into the sediment must be interpreted with caution because the Oyster ground sediment is known to be an area of intense bioturbation by macro- and meiobenthos even in periods of low oxygen concentration in the bottom water (De Wilde et al., 1984; Duineveld et al., 1991), thus the import of oxygen by macrofaunal burrows into the deeper sediment could be relevant (Kristensen, 2000; Meysman et al., 2006). A study by Laverock et al. (2010) has also shown that bioturbation can induce changes in the microbial community composition, resulting in distinct communities within the burrow. However, in our study we did not observe changes in the diversity of *amo*A gene sequences of AOA or AOB, which might indicate that the changing physicochemical conditions in this system do not select for specific ammonia oxidizing microorganisms. The depth and seasonal variability for AOB was more pronounced than for AOA in the first 4 cm of the sediment, which is consistent with the fact that the relative abundance of AOB is more significantly affected by bioturbation than AOA according to previous studies (Laverock et al., 2013).

AOA and AOB 16S rRNA and *amo*A genes and AOA-IPLs and were detected up to 12 cm depth in Oyster ground sediments. The detection of aerobic ammonia oxidizing microorganisms in marine sediment at depths where the oxygen concentration is expected to be undetectable seems counterintuitive with their aerobic metabolism. However, the intense bioturbation reported in the Oyster ground sediments, as mentioned above, might provide enough oxygen to support the activity of these microbial groups. In fact, Beman et al. (2012) detected AOB and AOA *amo*A genes up to 10 cm sediment depth as well as readily detectable ^15^NH_4_^+^ oxidation rates up to 9 cm bsf with maxima overlapping with a peak in pore water NO_3_^-^ + NO_2_^-^ concentrations between 3 and 7 cm bsf in bioturbated sediments of Catalina Island (CA, USA). Thus, our results, and those of Beman et al. (2012), suggest that aerobic ammonia oxidizers are indeed living and actively involved in the marine sedimentary nitrogen cycle deeper (i.e., up to at least ca. 10 cm) in coastal marine sediments.

The detection of a potential transcriptional activity of the AOA 16S rRNA gene up to 12 cm depth supports that AOA are indeed active deeper in the marine sediment. The potential AOA 16S rRNA gene transcriptional activity was higher during winter, whereas the abundance was slightly higher during summer months. Previous studies showed higher AOA abundance during winter months in the in the North Sea water column (Wuchter et al., 2006; Herfort et al., 2007; Pitcher et al., 2011c) attributed to the higher availability of ammonia and the lack of competition for ammonia with phytoplankton. However, none of these studies targeted the transcriptional activity of AOA. Recently, Bale et al. (2013) observed that the abundance of the benthic Thaumarchaeota (given by HPH crenarchaeol) in the surface sediment (0–1 cm) at the Oyster grounds was on average highest in the summer (August) and lowest in the winter (November), which they attributed to the deposition of algal-bloom-derived organic matter taking place in late spring and summer resulting in the formation of ammonia. However, it should also be noted that Bale et al. (2013) did find that transcriptional activity of the AOA 16S rRNA gene was higher during winter in the surface sediment. The disparity in seasonality between the results of these two studies highlights the difference between the seasonal ammonia dynamics at the surface sediment/water interface (Bale et al., 2013) and with depth in the sediment (this study).

To complement the determination of potential transcriptional activity of AOA based on 16S rRNA gene, we also determined the AOA *amo*A gene RNA:DNA ratio, which was possible for samples from August throughout the core but only in the upper 3 cm depth in February because the quantity of transcripts in the other seasons and depths was under the detection limit of the qPCR assay.

The relationship of *amo*A gene expression and *in situ* ammonia oxidation is still unclear (Lam et al., 2007; Nicol et al., 2008; Abell et al., 2011; Vissers et al., 2013). However, recent studies have observed a good correlation between nitrification rates and abundance of AOA *amo*A gene and transcripts in coastal waters (Smith et al., 2014; Urakawa et al., 2014). Therefore, the detection of AOA *amo*A transcripts in our setting suggests that AOA were active in the summer throughout the sediment in comparison with the winter in which AOA would be only active in the upper part of the sediment (upper 2 cm).

AOB 16S rRNA gene abundance was in the same order of magnitude than AOA 16S rRNA gene abundance; however, AOB *amoA* gene abundance was one order of magnitude lower than AOA *amo*A gene abundance, which can be explained by biases introduced by the primers or by differences in copy number of 16S rRNA and *amo*A genes per cell. In fact, it is assumed that 3.3 ± 1.6 copies of 16S rRNA can exist per cell in AOB (Větrovský and Baldrian, 2013), whereas marine Crenarchaeota seem to contain only one copy per genome (Klappenbach et al., 2001; Laverock et al., 2013). For the *amo*A gene it is assumed that AOB genomes contain two to three copies (Arp et al., 2007) while AOA genomes contain only one copy. In addition, the transcriptional activity of the AOB 16S rRNA gene was lower compared to the one of AOA 16S rRNA gene in all seasons.

On the other hand, the abundance of AOB *amo*A gene transcripts was higher than for AOA and was detected up to 12 cm depth in all seasons which would suggest a higher AOB activity vs. AOA. However, a recent study by Urakawa et al. (2014) in water of the Puget Sound Estuary did not observe a correlation between nitrification rates and AOB *amo*A transcripts. In addition, previous studies have detected the presence of AOB *amo*A transcripts in non-active starved cells (Bollmann et al., 2005; Geets et al., 2006; Sayavedra-Soto and Arp, 2011) indicating that the AOB *amo*A transcriptional activity is not a good indicator of AOB nitrification activity due to a longer half-life of AOB *amo*A transcripts than that expected for AOA *amo*A transcripts.

Taking together the AOA and AOB *amo*A gene quantification results, AOA have a more important role in the nitrification process in Oyster ground sediments than AOB. In addition, all available evidence points to the presence of some oxygen deeper in the sediments of the Oyster ground in all seasons probably due to the intense bioturbation in the area, which favors the activity of both AOB and AOA. In the case of AOA, and due to their higher affinity for oxygen (Martens-Habbena et al., 2009), the niche occupancy of AOA could be potentially larger than for AOB.

Previous studies observed a positive correlation between AOA abundance and low phosphate concentrations and suggested that phosphate plays a role in determining the niches of Thaumarchaeota (Erguder et al., 2009; Dang et al., 2013). In our study, AOA *amo*A gene transcript copy number and RNA:DNA ratio was negatively correlated with phosphate (*r*_s_ = -0.745 and -0.739, respectively; both *P* ≤ 0.005) suggesting a lower expression of the AOA *amo*A gene with an increase in pore water phosphate concentrations. These results support the assumption that some ecotypes of AOA dominate over AOB in environments with low phosphate availability. Previously, homologs of the *pst* gene, encoding for the Pst phosphate ABC transporter, which is usually activated in phosphate deficient environments, was found in marine metagenomes of thaumarchaeotal origin (Rusch et al., 2007; Dang et al., 2013) emphasizing the importance of phosphate availability in marine environments. No correlations were found between AOB abundance or transcriptional activity with pore water nitrogen species, but a negative correlation with the sediment depth (*r*_s_ = –0.646; *P* ≤ 0.005) was observed indicating that sediment depth is a factor determining the AOB abundance and activity in Oyster ground sediments which might be explained by the decreasing oxygen availability with depth.

### ANAEROBIC AMMONIA OXIDIZERS

Anammox bacteria 16S rRNA and *hzs*A gene abundance was detected throughout the analyzed sediment with more pronounced depth and seasonal differences than those observed for AOA and AOB. This was not reflected in the abundance of PC-monoether ladderane lipid. It is possible that the PC-monoether ladderane is partly derived from fossil material and thus reflects a long-term presence of anammox bacteria averaged throughout the entire year rather than an actively living anammox population. This possibility has been already suggested by Brandsma et al. (2011) and Bale et al. (2014) who found dissimilarities between the PC-monoether ladderane concentration and gene copy numbers in surface sediments of the Gullmar Fjord and the Southern North Sea, respectively.

We also observed an up to 10-fold difference in the quantification of anammox bacteria 16S rRNA and *hzs*A gene abundance (**Figures 4A,B**). This discordance between the anammox bacteria gene markers has been previously observed in environmental samples (Harhangi et al., 2012), and it could be due to primer biases as previously suggested (Bale et al., 2014). Both the anammox 16S rRNA and the *hzs*A gene revealed a higher abundance in August compared to February and May. This may indicate that anammox bacteria are more active and abundant at higher temperatures (15°C), which is the anammox bacteria temperature optimum in temperate shelf sediments (Dalsgaard et al., 2005). Additionally, the oxygen penetration depth is expected to decrease during water stratification between May and October, which would also favor anammox bacterial growth and activity (Dalsgaard and Thamdrup, 2002). Previous studies by Neubacher et al. (2013) observed an increase in anammox activity up to 82% compared to control treatments in mesocosms under sustained hypoxic conditions, which corresponds well with our findings of high anammox activity during summer stratification associated with lower oxygen concentrations in the bottom water (Figure S1 in Supplementary Materials).

The presence of anammox markers in the upper layers of the sediment (0–4 cm) is remarkable as the metabolism of anammox bacteria is expected to be incompatible with the presence of oxygen. However, some studies have reported that anammox bacteria can cope with low oxygen concentrations (Kalvelage et al., 2011; Yan et al., 2012), and that anammox bacteria can be present and active at high oxygen levels by being dormant or using anaerobic niches (Kuypers et al., 2005; Woebken et al., 2007). It is also possible that a high activity of AOA and AOB and heterotrophic bacteria inhabiting these marine sediments would rapidly consume the oxygen available and allow the activity of anammox bacteria even in presence of oxygen. Some studies have also concluded that the potential for anammox bacteria activity is indeed not affected by bioturbation or physical mixing (Rooks et al., 2012). On the contrary, bioturbation and mixing can extend the area of nitrate reduction and thus the availability of nitrite, which fuels the anammox process (Meyer et al., 2005). Our results thus suggest that anammox bacteria can tolerate the presence of oxygen and co-exist with aerobic ammonia oxidizers and potentially have an important role in the nitrogen cycle in marine sediments. In fact, the potential transcriptional activity of anammox bacteria 16S rRNA and *hzs*A gene was observed throughout the sediment in all seasons. The transcriptional activity of anammox bacteria 16S rRNA gene was higher in the summer as observed for the anammox bacteria abundance. A recent study by Bale et al. (2014) observed a good correlation between the rate of anammox and anammox bacteria 16S rRNA gene transcript abundance in North Sea sediments, which suggests that the transcriptional activity of anammox bacteria 16S rRNA gene is a good proxy of anammox bacteria activity, and confirm a higher anammox activity in the summer for the sediments analyzed in our study. Nevertheless, quantification results of anammox bacteria 16S rRNA gene transcripts have to be interpret with caution because there is evidence that the ribosome content does not decrease during the period of starvation (Schmid et al., 2005; Li and Gu, 2011). However, the increase of anammox bacteria 16S rRNA gene transcriptional activity during summer was not observed for the *hzs*A gene, which remained stable across sediment depth and also for the different seasons, and gene abundances were generally an order of magnitude lower. The low and constitutive transcriptional activity of the *hzs*A gene indicates that the *hzs*A gene transcript abundance does not reflect variations of anammox bacteria activity in environmental sediment samples, thus the *hzs*A gene transcript abundance does not seem to be an adequate biomarker for the estimation of the activity of anammox bacteria. Further experiments, especially with pure cultures and under controlled physiological conditions, should be performed to clarify the regulation of the expression of the *hzs*A gene.

No evidence of a niche separation of aerobic and anaerobic ammonia oxidizers was observed in the oxygen transition zone of the marine sediments. Surprisingly, aerobic and anaerobic ammonia oxidizers are present and active throughout the year in oxygenated sediment layers as well as in anoxic layers. These results seem to be counterintuitive with regards to the individual oxygen requirements of the targeted microbial groups. We hypothesize that the presence and activity of aerobic ammonia oxidizers (AOA, AOB) is supported by the oxygen supply in deeper anoxic layers due to bioturbation. Likewise, the presence and activity of anammox bacteria in these sediments is then possible by the rapid consumption of oxygen by aerobic ammonia oxidizers and/or other groups, as well as the availability of nitrite provided either by ammonia oxidizers or by an active nitrate reduction present in bioturbated sediments (Meyer et al., 2005).

## CONCLUSION

Our study has unraveled the coexistence and metabolic activity of AOA, AOB, and anammox bacteria in bioturbated marine sediments of the North Sea, leading to the conclusion that the metabolism of these microbial groups is spatially coupled based on the rapid consumption of oxygen that allows the coexistence of aerobic and anaerobic ammonia oxidizers. AOA outnumbered AOB throughout the year which may be caused by the higher oxygen affinity of AOA compared to AOB. Anammox bacterial abundance and activity were higher during summer, indicating that their growth and activity are favored by higher temperatures and lower oxygen available in the sediments due to summer stratifying conditions in the water column. During the summer, anammox bacteria are probably not in competition with AOA and AOB for ammonia as the concentrations were relatively high in the sediment pore water most likely as a result of ammonification processes and the activity of denitrifiers and DNRA. Further studies are required to get a complete picture of the nature of these interactions in the oxygen transition zone of coastal marine sediments.

## Conflict of Interest Statement

The authors declare that the research was conducted in the absence of any commercial or financial relationships that could be construed as a potential conflict of interest.

## References

[B1] Abell G. C. J. Banks J. Ross D. J. Keane J. P. Robert S. S. Revill A. T. (2011). Effects of estuarine sediment hypoxia on nitrogen fluxes and ammonia oxidizer gene transcription. *FEMS Microbiol. Ecol.* 75 111–122 10.1111/j.1574-6941.2010.00988.x 21083579

[B2] Abell G. C. J. Revill A. T. Smith C. Bissett A. P. Volkman J. K. Robert S. S. (2010). Archaeal ammonia oxidizers and nirS-type denitrifiers dominate sediment nitrifying and denitrifying populations in a subtropical macrotidal estuary. *ISME J.* 4 286–300 10.1038/ismej.2009.105 19798039

[B3] Arp D. J. Chain P. S. G. Klotz M. G. (2007). The impact of genome analyses on our understanding of ammonia-oxidising bacteria. *Annu. Rev. Microbiol.* 61 503–528 10.1146/annurev.micro.61.080706.093449 17506671

[B4] Bale N. J. Villanueva L. Fan H. Stal L. J. Hopmans E. C. Schouten S. (2014). Occurrence and activity of anammox bacteria in surface sediments of the southern North Sea. *FEMS Microbiol. Ecol.* 89 99–110 10.1111/1574-6941.12338 24716573

[B5] Bale N. J. Villanueva L. Hopmans E. C. Schouten S. Sinninghe Damsté J. S. (2013). Different seasonality of pelagic and benthic Thaumarchaeota in the North Sea. *Biogeoscience* 10 7195–7206 10.5194/bg-10-7195-2013

[B6] Beman J. M. Bertics V. J. Braunschweiler T. Wilson J. M. (2012). Quantification of ammonia oxidation rates and the distribution of ammonia-oxidizing Archaea and bacteria in marine sediment depth profiles from Catalina Island, California. *Front. Microbiol.* 3:263 10.3389/fmicb.2012.00263 PMC3403348 22837756

[B7] Beman J. M. Francis C. A. (2006). Diversity of ammonia-oxidizing Archaea and bacteria in the sediments of a hypernutrified subtropical estuary: Bahía del Tóbari, Mexico. *Appl. Environ. Microbiol.* 72 7767–7777 10.1128/AEM.00946-06 17012598 PMC1694203

[B8] Bernhard A. E. Landry Z. C. Blevins A. de la Torre J. R. Giblin A. E. Stahl D. A. (2010). Abundance of ammonia-oxidizing Archaea and bacteria along an estuarine salinity gradient in relation to potential nitrification rates. *Appl. Environ. Microbiol.* 76 1285–1289 10.1128/AEM.02018-09 20038706 PMC2820943

[B9] Bollmann A. Schmidt I. Saunders A. M. Nicolaisen M. H. (2005). Influence of starvation on potential ammonia-oxidizing activity and amoA mRNA levels of *Nitrosospira briensis*. *Appl. Environ. Microbiol.* 71 1276–1282 10.1128/AEM.71.3.1276-1282.2005 15746329 PMC1065156

[B10] Brandsma J. van de Vossenberg J. Risgaard-Petersen N. Schmid M. C. Engström P. Eurenius K. (2011). A multi-proxy study of anaerobic ammonium oxidation in marine sediments of the Gullmar Fjord, Sweden. *Environ. Microbiol. Rep.* 3 360–366 10.1111/j.1758-2229.2010.00233.x 23761282

[B11] Brochier-Armanet C. Boussau B. Gribaldo S. Forterre P. (2008). Mesophilic Crenarchaeota: proposal for a third archaeal phylum, the Thaumarchaeota. *Nat. Rev. Microbiol.* 6 245–252 10.1038/nrmicro1852 18274537

[B12] Coolen M. J. L. Abbas B. van Bleijswijk J. Hopmans E. C. Kuypers M. M. M. Wakeham S. G. (2007). Putative ammonia-oxidizing Crenarchaeota in suboxic waters of the Black Sea: a basin-wide ecological study using 16S ribosomal and functional genes and membrane lipids. *Environ. Microbiol.* 9 1001–1016 10.1111/j.1462-2920.2006.01227.x 17359272

[B13] Dale O. R. Tobias C. R. Song B. (2009). Biogeographical distribution of diverse anaerobic ammonium oxidizing (anammox) bacteria in Cape Fear River Estuary. *Environ. Microbiol.* 11 1194–1207 10.1111/j.1462-2920.2008.01850.x 19161435

[B14] Dalsgaard T. Thamdrup B. (2002). Factors controlling anaerobic ammonium oxidation with nitrite in marine sediments. *Appl. Environ. Microbiol.* 68 3802–3808 10.1128/AEM.68.8.3802-3808.2002 12147475 PMC124030

[B15] Dalsgaard T. Thamdrup B. Canfield D. E. (2005). Anaerobic ammonium oxidation (anammox) in the marine environment. *Res. Microbiol.* 156 457–464 10.1016/j.resmic.2005.01.011 15862442

[B16] Dang H. Chen R. Wang L. Guo L. Chen P. Tang Z. (2010). Environmental factors shape sediment anammox bacterial communities in hypernutrified Jiaozhou Bay, China. *Appl. Environ. Microbiol.* 76 7036–7047 10.1128/AEM.01264-10 20833786 PMC2976235

[B17] Dang H. Zhou H. Yang J. Ge H. Jiao N. Luan X. (2013). Thaumarchaeotal signature gene distribution in sediments of the northern South China Sea: an indicator of the metabolic intersection of the marine carbon, nitrogen, and phosphorus cycles? *Appl. Environ. Microbiol.* 79 2137–2147 10.1128/AEM.03204-12 23335759 PMC3623242

[B18] De Wilde P. A. W. J. Berghuis E. M. Kok A. (1984). Structure and energy demand of the benthic community of the Oyster Ground, central North Sea. *Netherlands J. Sea Res.* 18 143–159 10.1016/0077-7579(84)90029-2

[B19] Duineveld G. C. A. Konitzer A. Niermann U. De Wilde P. A. W. J. Gray J. S. (1991). The macrobenthos of the North Sea. *Netherlands J. Sea Res.* 28 53–65 10.1016/0077-7579(91)90004-K

[B20] Engström P. Dalsgaard T. Hulth S. Aller R. C. (2005). Anaerobic ammonium oxidation by nitrite (anammox): implications for N_2_ production in coastal marine sediments. *Geochim. Cosmochim. Acta* 69 2057–2065 10.1016/j.gca.2004.09.032

[B21] Erguder T. H. Boon N. Wittebolle L. Marzorati M. Verstraete W. (2009). Environmental factors shaping the ecological niches of ammonia-oxidizing archaea. *FEMS Microbiol. Rev.* 33 855–869 10.1111/j.1574-6976.2009.00179.x 19453522

[B22] Geets J. Boon N. Verstraete W. (2006). Strategies of aerobic ammonia-oxidizing bacteria for coping with nutrient and oxygen fluctuations. *FEMS Microbiol. Ecol.* 58 1–13 10.1111/j.1574-6941.2006.00170.x 16958903

[B23] Greenwood N. Parker E. R. Fernand L. Sivyer D. B. Weston K. Painting S. J. (2010). Detection of low bottom water oxygen concentrations in the North Sea: implications for monitoring and assessment of ecosystem health. *Biogeoscience* 7 1357–1373 10.5194/bg-7-1357-2010

[B24] Gruber N. Galloway J. N. (2008). An Earth-system perspective of the global nitrogen cycle. *Nature* 451 293–296 10.1038/nature06592 18202647

[B25] Harhangi H. R. Le Roy M. van Alen T. Hu B.-L. Groen J. Kartal B. (2012). Hydrazine synthase, a unique phylomarker with which to study the presence and biodiversity of anammox bacteria. *Appl. Environ. Microbiol.* 78 752–758 10.1128/AEM.07113-11 22138989 PMC3264106

[B26] Herfort L. Schouten S. Abbas B. Veldhuis M. J. W. Coolen M. J. L. Wuchter C. (2007). Variations in spatial and temporal distribution of Archaea in the North Sea in relation to environmental variables. *FEMS Microbiol. Ecol.* 62 242–257 10.1111/j.1574-6941.2007.00397.x 17991018

[B27] Holmes D. E. Nevin K. P. Lovley D. R. Holmes E. T. (2004). In situ expression of nifD in Geobacteraceae in subsurface sediments. *Appl. Environ. Microbiol*. 70 7251–7259 10.1128/AEM.70.12.7251-7259.2004 15574924 PMC535187

[B28] Hornek R. Pommerening-Röser A. Koops H.-P. Farnleitner A. H. Kreuzinger N. Kirschner A. (2006). Primers containing universal bases reduce multiple amoA gene specific DGGE band patterns when analyzing the diversity of beta-ammonia oxidizers in the environment. *J. Microbiol. Methods* 66 147–155 10.1016/j.mimet.2005.11.001 16343671

[B29] Jaeschke A. Rooks C. Trimmer M. Nicholls J. C. Hopmans E. C. Schouten S. (2009). Comparison of ladderane phospholipid and core lipids as indicators for anaerobic ammonium oxidation (anammox) in marine sediments. *Geochim. Cosmochim. Acta* 73 2077–2088 10.1016/j.gca.2009.01.013

[B30] Jukes T. H. Cantor C. R. (1969). “Evolution of protein molecules,” in *Mammalian Protein Metabolism* ed. Munro H. N. (New York: Academic Press) 21–132

[B31] Kalvelage T. Jensen M. M. Contreras S. Revsbech N. P. Lam P. Günter M. (2011). Oxygen sensitivity of anammox and coupled N-cycle processes in oxygen minimum zones. *PLoS ONE* 6:e29299 10.1371/journal.pone.0029299 PMC3247244 22216239

[B32] Klappenbach J. A. Saxman P. R. Cole J. R. Schmidt T. M. (2001) rrndb: the ribosomal RNA operon copy number database. *Nucleic Acids Res.* 29 181–184 10.1093/nar/29.1.181 11125085 PMC29826

[B33] Könneke M. Bernhard A. E. de la Torre J. R. Walker C. B. Waterbury J. B. Stahl D. A. (2005). Isolation of an autotrophic ammonia-oxidizing marine archaeon. *Nature* 437 543–546 10.1038/nature03911 16177789

[B34] Kowalchuk G. A. Stephen J. R. De Boer W. Prosser J. I. Embley T. M. Woldendorp J. W. (1997). Analysis of ammonia-oxidizing bacteria of the beta subdivision of the class *Proteobacteria* in coastal sand dunes by denaturing gradient gel electrophoresis and sequencing of PCR-amplified 16S ribosomal DNA fragments. *Appl. Environ. Microbiol*. 63 1489–1497 9097446 10.1128/aem.63.4.1489-1497.1997 PMC168443

[B35] Kristensen E. (2000). Organic matter diagenesis at the oxic/anoxic interface in coastal marine sediments, with emphasis on the role of burrowing animals. *Hydrobiologia* 426 1–24 10.1023/A:1003980226194

[B36] Kuenen J. G. (2008). Anammox bacteria: from discovery to application. *Nat. Rev. Microbiol.* 6 320–326 10.1038/nrmicro1857 18340342

[B37] Kuypers M. M. M. Lavik G. Woebken D. Schmid M. Fuchs B. M. Amann R. (2005). Massive nitrogen loss from the Benguela upwelling system through anaerobic ammonium oxidation. *Proc. Natl. Acad. Sci. U.S.A.* 102 6478–6483 10.1073/pnas.0502088102 15843458 PMC556276

[B38] Kuypers M. M. M. Sliekers A. O. Lavik G. Schmid M. Jørgensen B. B. Kuenen J. G. (2003). Anaerobic ammonium oxidation by anammox bacteria in the Black Sea. *Nature* 422 2–5 10.1038/nature01472 12686999

[B39] Lam P. Jensen M. M. Lavik G. McGinnis D. F. Müller B. Schubert C. J. (2007). Linking crenarchaeal and bacterial nitrification to anammox in the Black Sea. *Proc. Natl. Acad. Sci. U.S.A.* 104 7104–7109 10.1073/pnas.0611081104 17420469 PMC1849958

[B40] Laverock B. Smith C. Tait K. Osborn A. M. Widdicombe S. Gilbert J. A. (2010). Bioturbating shrimp alter the structure and diversity of bacterial communities in coastal marine sediments. *ISME J.* 4 1531–1544 10.1038/ismej.2010.86 20596074

[B41] Laverock B. Tait K. Gilbert J. A. Osborn A. M. Widdicombe S. (2013). Impacts of bioturbation on temporal variation in bacterial and archaeal nitrogen-cycling gene abundance in coastal sediments. *Environ. Microbiol. Rep.* 6 113–121 10.1111/1758-2229.12115 24596269 PMC4208606

[B42] Li M. Gu J.-D. (2011). Advances in methods for detection of anaerobic ammonium oxidizing (anammox) bacteria. *Appl. Microbiol. Biotechnol*. 90 1241–1252 10.1007/s00253-011-3230-6 21476137 PMC3082692

[B43] Li M. Hong Y. Klotz M. G. Gu J.-D. (2010). A comparison of primer sets for detecting 16S rRNA and hydrazine oxidoreductase genes of anaerobic ammonium-oxidizing bacteria in marine sediments. *Appl. Microbiol. Biotechnol.* 86 781–790 10.1007/s00253-009-2361-5 20107988

[B44] Lohse L. Epping E. Helder W. van Raaphorst W. (1996). Oxygen pore water profiles in continental shelf sediments of the North Sea: turbulent versus molecular diffusion. *Mar. Ecol. Prog. Ser.* 145 63–75 10.3354/meps145063

[B45] Ludwig W. Strunk O. Westram R. Richter L. Meier H. Yadhukumar. (2004). ARB: a software environment for sequence data. *Nucleic Acids Res.* 32 1363–1371 10.1093/nar/gkh293 14985472 PMC390282

[B46] Martens-Habbena W. Berube P. M. Urakawa H. de la Torre J. R. Stahl D. A. (2009). Ammonia oxidation kinetics determine niche separation of nitrifying Archaea and bacteria. *Nature* 461 976–979 10.1038/nature08465 19794413

[B47] Meyer R. L. Risgaard-Petersen N. Allen D. E. (2005). Correlation between Anammox activity and microscale distribution of nitrite in a subtropical mangrove sediment. *Appl. Environ. Microbiol.* 71 6142–6149 10.1128/AEM.71.10.6142-6149.2005 16204532 PMC1265924

[B48] Meysman F. J. R. Middelburg J. J. Heip C. H. R. (2006). Bioturbation: a fresh look at Darwin’s last idea. *Trends Ecol. Evol.* 21 688–695 10.1016/j.tree.2006.08.002 16901581

[B49] Mosier A. C. Francis C. A. (2008). Relative abundance and diversity of ammonia-oxidizing Archaea and bacteria in the San Francisco Bay estuary. *Environ. Microbiol.* 10 3002–3016 10.1111/j.1462-2920.2008.01764.x 18973621

[B50] Neubacher E. C. Parker R. E. Trimmer M. (2011). Short-term hypoxia alters the balance of nitrogen cycle in coastal sediments *.Limnol. Oceanogr*. 56 651–665 10.4319/lo.2011.56.2.0651

[B51] Neubacher E. C. Parker R. E. Trimmer M. (2013). The potential effect of sustained hypoxia on nitrogen cycling in sediment from the southern North Sea: a mesocosm experiment. *Biogeochemistry* 113 69–84 10.1007/s10533-012-9749-5

[B52] Nicholls J. Trimmer M. (2009). Widespread occurrence of the Anammox reaction in estuarine sediments. *Aquat. Microb. Ecol.* 55 105–113 10.3354/ame01285

[B53] Nicol G. W. Leininger S. Schleper C. Prosser J. I. (2008). The influence of soil pH on the diversity, abundance and transcriptional activity of ammonia oxidizing Archaea and bacteria. *Environ. Microbiol.* 10 2966–2978 10.1111/j.1462-2920.2008.01701.x 18707610

[B54] Pester M. Rattei T. Flechl S. Gröngröft A. Richter A. Overmann J. (2012). amoA-based consensus phylogeny of ammonia-oxidizing Archaea and deep sequencing of amoA genes from soils of four different geographic regions. *Environ. Microbiol.* 14 525–539 10.1111/j.1462-2920.2011.02666.x 22141924 PMC3328746

[B55] Pitcher A. Hopmans E. C. Mosier A. C. Park S.-J. Rhee S.-K. Francis C. A. (2011a). Core and intact polar glycerol dibiphytanyl glycerol tetraether lipids of ammonia-oxidizing Archaea enriched from marine and estuarine sediments. *Appl. Environ. Microbiol.* 77 3468–3477 10.1128/AEM.02758-10 21441324 PMC3126447

[B56] Pitcher A. Villanueva L. Hopmans E. C. Schouten S. Reichart G.-J. Sinninghe Damsté J. S. (2011b). Niche segregation of ammonia-oxidizing archaea and anammox bacteria in the Arabian Sea oxygen minimum zone. *ISME J.* 5 1896–1904 10.1038/ismej.2011.60 21593795 PMC3223301

[B57] Pitcher A. Wuchter C. Siedenberg K. Schouten S. Sinninghe Damsté J. S. (2011c). Crenarchaeol tracks winter blooms of ammonia-oxidizing Thaumarchaeota in the coastal North Sea. *Limnol. Oceanogr.* 56 2308–2318 10.4319/lo.2011.56.6.2308

[B58] Prosser J. I. Nicol G. W. (2008). Relative contributions of archaea and bacteria to aerobic ammonia oxidation in the environment. *Environ. Microbiol*. 10 2931–2941 10.1111/j.1462-2920.2008.01775.x 18973620

[B59] Raaphorst W. van Malschaert H. van Haren H. (1998). Tidal resuspension and deposition of particulate matter in the Oyster Grounds, North Sea. *J. Mar. Res.* 56 257–291 10.1357/002224098321836181

[B60] Rooks C. Schmid M. C. Mehsana W. Trimmer M. (2012). The depth-specific significance and relative abundance of anaerobic ammonium-oxidizing bacteria in estuarine sediments (Medway Estuary, UK). *FEMS Microbiol. Ecol.* 80 19–29 10.1111/j.1574-6941.2011.01266.x 22133008

[B61] Rotthauwe J. H. Witzel K. P. Liesack W. (1997). The ammonia monooxygenase structural gene amoA as a functional marker: molecular fine-scale analysis of natural ammonia-oxidizing populations. *Appl. Environ. Microbiol.* 63 4704–4712 9406389 10.1128/aem.63.12.4704-4712.1997 PMC168793

[B62] Rusch D. B. Halpern A. L. Sutton G. Heidelberg K. B. Williamson S. Yooseph S. (2007). The Sorcerer II Global Ocean Sampling expedition: northwest Atlantic through eastern tropical Pacific. *PLoS Biol.* 5:e77 10.1371/journal.pbio.0050077 PMC1821060 17355176

[B63] Sahan E. Muyzer G. (2008). Diversity and spatio-temporal distribution of ammonia-oxidizing Archaea and bacteria in sediments of the Westerschelde estuary. *FEMS Microbiol. Ecol.* 64 175–186 10.1111/j.1574-6941.2008.00462.x 18336555

[B64] Saitou N. Nei M. (1987). The neighbor-joining method: a new method for reconstructing phylogenetic trees. *Mol. Biol. Evol.* 4 406–425 3447015 10.1093/oxfordjournals.molbev.a040454

[B65] Sakami T. (2012). Distribution of ammonia-oxidizing Archaea and bacteria in the surface sediments of Matsushima Bay in relation to environmental variables. *Microbes Environ.* 27 61–66 10.1264/jsme2.ME11218 22200641 PMC4036025

[B66] Santoro A. E. Francis C. A. De Sieyes N. R. Boehm A. B. (2008). Shifts in the relative abundance of ammonia-oxidizing bacteria and Archaea across physicochemical gradients in a subterranean estuary. *Environ. Microbiol.* 10 1068–1079 10.1111/j.1462-2920.2007.01547.x 18266758

[B67] Sayavedra-Soto L. A. Arp D. J. (2011). “Ammonia-oxidizing bacteria: their biochemistry and molecular biology,” in *Nitrification* eds Ward B. B. Arp D. J. Klotz M. (Washington, DC: ASM Press) 11–37

[B68] Schmid M. C. Maas B. Dapena A. Van De Pas-Schoonen K. Van De Vossenberg J. Kartal B. (2005). Minireview: biomarkers for in situ detection of anaerobic ammonium-oxidizing (Anammox) bacteria. *Appl. Environ. Microbiol*. 71 1677–1684 10.1128/AEM.71.4.1677-1684.2005 15811989 PMC1082507

[B69] Schouten S. Hopmans E. C. Baas M. Boumann H. Standfest S. Könneke M. (2008). Intact membrane lipids of “Candidatus Nitrosopumilus maritimus,” a cultivated representative of the cosmopolitan mesophilic group I *Crenarchaeota*. *Appl. Environ. Microbiol.* 74 2433–2440 10.1128/AEM.01709-07 18296531 PMC2293165

[B70] Sinninghe Damsté J. S. Schouten S. Hopmans E. C. van Duin A. C. T. Geenevasen J. A. G. (2002a). Crenarchaeol: the characteristic core glycerol dibiphytanyl glycerol tetraether membrane lipid of cosmopolitan pelagic Crenarchaeota. *J. Lipid Res.* 43 1641–1651 10.1194/jlr.M200148-JLR200 12364548

[B71] Sinninghe Damsté J. S. Strous M. Rijpstra W. I. C. Geenevasen J. A. G. van Duin A. C. T. van Niftrik L. A. (2002b). Linearly concatenated cyclobutane lipids form a dense bacterial membrane. *Nature* 419 8–12 10.1038/nature01128 12384695

[B72] Smith J. M. Cascotti K. L. Chavez F. P. Francis C. (2014). Differential contributions of archaeal ammonia oxidizer ecotypes to nitrification in coastal surface waters. *ISME J*. 8 1704–1714 10.1038/ismej.2014.11 24553472 PMC4817602

[B73] Strous M. Kuenen J. G. Jetten M. S. (1999). Key physiology of anaerobic ammonium oxidation. *Appl. Environ. Microbiol.* 65 3248–3250 10388731 10.1128/aem.65.7.3248-3250.1999 PMC91484

[B74] Tamura K. Peterson D. Peterson N. Stecher G. Nei M. Kumar S. (2011). MEGA5: molecular evolutionary genetics analysis using maximum likelihood, evolutionary distance, and maximum parsimony methods. *Mol. Biol. Evol.* 28 2731–2739 10.1093/molbev/msr121 21546353 PMC3203626

[B75] Thamdrup B. Dalsgaard T. (2002). Production of N_2_ through anaerobic ammonium oxidation coupled to nitrate reduction in marine sediments. *Appl. Environ. Microbiol.* 68 1312–1318 10.1128/AEM.68.3.1312-1318.2002 11872482 PMC123779

[B76] Urakawa H. Martens-Habbena W. Huguet C. de la Torre J. R. Ingalls A. E. Devol A. H. (2014). Ammonia availability shapes the seasonal distribution and activity of archaeal and bacterial ammonia oxidizers in the Puget Sound Estuary. *Limnol. Oceanogr*. 59 1321–1335 10.4319/lo.2014.59.4.1321

[B77] van Raaphorst W. Kloosterhuis H. T. Berghuis E. M. Gieles A. J. M. van Noort G. J. (1992). Nitrogen cycling in two types of sediments of the southern North Sea (Frisian front, Broad Fourteens): field data and mesocosm results. *Netherlands J. Sea Res.* 28 293–316 10.1016/0077-7579(92)90033-B

[B78] Větrovský T. Baldrian P. (2013). The variability of the 16S rRNA gene in bacterial genomes and its consequences for bacterial community analyses. *PLoS ONE* 8:e57923 10.1371/journal.pone.0057923 PMC3583900 23460914

[B79] Vissers E. W. Anselmetti F. S. Bodelier P. L. E. Muyzer G. Schleper C. Tourna M. (2013). Temporal and spatial coexistence of archaeal and bacterial amoA genes and gene transcripts in Lake Lucerne. *Archaea* 2013: Article ID 289478, 11 p. 10.1155/2013/289478 PMC3603158 23533328

[B80] Weston K. Fernand L. Nicholls J. Marca-Bell A. Mills D. Sivyer D. (2008). Sedimentary and water column processes in the Oyster Grounds: a potentially hypoxic region of the North Sea. *Mar. Environ. Res.* 65 235–249 10.1016/j.marenvres.2007.11.002 18082251

[B81] Woebken D. Fuchs B. M. Kuypers M. M. M. Amann R. (2007). Potential interactions of particle-associated anammox bacteria with bacterial and archaeal partners in the Namibian upwelling system. *Appl. Environ. Microbiol.* 73 4648–4657 10.1128/AEM.02774-06 17526789 PMC1932835

[B82] Woebken D. Lam P. Kuypers M. M. M. Naqvi S. W. A. Kartal B. Strous M. (2008). A microdiversity study of anammox bacteria reveals a novel Candidatus Scalindua phylotype in marine oxygen minimum zones. *Environ. Microbiol.* 10 3106–3119 10.1111/j.1462-2920.2008.01640.x 18510553

[B83] Wuchter C. Abbas B. Coolen M. J. L. Herfort L. van Bleijswijk J. Timmers P. (2006). Archaeal nitrification in the ocean. *Proc. Natl. Acad. Sci. U.S.A.* 103 12317–12322 10.1073/pnas.0600756103 16894176 PMC1533803

[B84] Yakimov M. M. La Cono V. Smedile F. De Luca T. H. Juárez S. Ciordia S. (2011). Contribution of crenarchaeal autotrophic ammonia oxidizers to the dark primary production in Tyrrhenian deep waters (Central Mediterranean Sea). *ISME J.* 5 945–961 10.1038/ismej.2010.197 21209665 PMC3131861

[B85] Yan J. Haaijer S. C. M. Op den Camp H. J. M. van Niftrik L. Stahl D. A. Könneke M. (2012). Mimicking the oxygen minimum zones: stimulating interaction of aerobic archaeal and anaerobic bacterial ammonia oxidizers in a laboratory-scale model system. *Environ. Microbiol.* 14 3146–3158 10.1111/j.1462-2920.2012.02894.x 23057688 PMC3558802

[B86] Zhang X. Agogué H. Dupuy C. Gong J. (2013). Relative abundance of ammonia oxidizers, denitrifiers, and Anammox bacteria in sediments of hyper-nutrified estuarine tidal flats and in relation to environmental conditions. *Clean* 41 1–9

