# Supplementary material for: Seasonality and depth distribution of the abundance and activity of ammonia oxidizing microorganisms in marine coastal sediments (North Sea)
Source: Front Microbiol. 2014 Sep 5;5:472. doi: 10.3389/fmicb.2014.00472 (PMC4155873; doi:10.3389/fmicb.2014.00472)
Supplement: Supplementary file 1 [file Presentation_1.PDF]

Supplementary material

Seasonality and depth distribution of the abundance and activity of ammonia oxidizing microorganisms in marine coastal sediments (North Sea)

Yvonne A. Lipsewers*, Nicole J. Bale, Ellen C. Hopmans, Stefan Schouten, Jaap S. Sinninghe Damsté, and Laura Villanueva

NIOZ Royal Netherlands Institute for Sea Research, Department of Marine Organic Biogeochemistry, PO Box 59, 1790AB Den Burg, The Netherlands

Table S1: Primer pairs described in the text, polymerase chain reaction (PCR) conditions and amplicon size used in this study.

Assay	Target	Primer pair	T_m	amplicon size [bp*]	Reference
qPCR	AOA 16S rRNA gene	MCGI-391F (5'-AAGGTTARTCCGAGTGRTTTC-3')<br>MCGI-554R (5'-TGACCACTTGAGGTGCTG-3')	qPCR 61 ˚C	163	1
qPCR + PCR/cloning	AOA *amo*A gene	AmoA-ModF (5'-GGTCTGGYTWAGACGATG-3')<br>AmoA-ModR (5'-GCCATCCATCTGTAWGTC-3')	qPCR 59.5 ˚C<br>PCR 55 ˚C	626	2
qPCR + PCR/cloning	Anammox bacteria 16S rRNA gene	Brod541F (5'-GAGCACGTAGGTGGGTTTGT-3')<br>Amx820R (5'-AAAACCCCTCTACTTAGTGCCC-3')	qPCR 59 ˚C<br>PCR 58 ˚C	279	3
qPCR	Anammox bacteria *hzs*A gene	*hzs*A_1597F (5'-WTYGGKTATCARTATGTA-3')<br>*hzs*A_1857R (5'-AAABGGYGAATCATARTGGC-3')	qPCR 51 ˚C	260	4
qPCR	AOB 16S rRNA gene	CTO189F_A (5'-GGAGRAAAGCAGGGGATCG-3')[†]<br>CTO189F_C (5'-GGAGGAAAGTAGGGGATCG-3')[†]<br>CTO654R (5'-CTAGCYTTGTAGTTTCAAACGC-3')	qPCR 60 ˚C	465	5
qPCR + PCR/cloning	AOB *amo*A gene	AOB-amoAF (5'-GGGGTTTCTACTGGTGGT-3')<br>AOB-amoAR new (5'-CCCCTCBGSAAAVCCTTCTTC-3')	qPCR 61.6 ˚C<br>PCR 58 ˚C	491	6, 7

PCR conditions: 95˚C 5 min; 40 × [95˚C 1 min, melting temperature (T_m) 40 s, 72˚C 1 min]; 72˚C 5 min; qPCR conditions: 95˚C 4 min; 40 × [95˚C 30 s, T_m 40 s, 72˚C 30 s]; 80˚C 25 s; [*]base pairs (bp); [†] The forward primer contains an equimolar mixture of the reported forward primers.

References:

1. Coolen et al. (2007)
2. Yakimov et al. (2011)
3. Li et al. (2010)
4. Harhangi et al. (2012)
5. Kowalchuk et al. (1997)
6. Rotthauwe et al. (1997)
7. Hornek et al. (2006)

Table S2: Quantitative polymerase chain reaction (qPCR) efficiencies (E) and correlation coefficients (R^2).

		February			May			August		
**qPCR assay**	**target molecule**	**T_m [˚C]**	**E [%]**	**R^2**	**T_m [˚C]**	**E [%]**	**R^2**	**T_m [˚C]**	**E [%]**	**R^2**
AOA 16S rRNA gene	DNA	61.0	100.0	0.998	61.0	93.5	0.988	61.0	94.3	0.999
(MCGI-391F/MCGI-554R)	cDNA	61.0	96.1	1.000	61.0	98.8	0.998	61.0	97.2	0.999
AOA *amo*A gene	DNA	59.5	100.7	0.998	59.5	99.7	0.988	59.5	97.8	0.994
(AmoA_modF/AmoA_modR)	cDNA	59.5	99.9	0.983	59.5	107.6	0.984	59.5	96.4	0.995
Anammox bacteria 16S rRNA gene	DNA	59.0	99.0	0.998	59.0	96.4	0.995	59.0	98.9	0.993
(Brod541F/Amx820R)	cDNA	59.0	98.4	0.999	59.0	93.7	0.996	59.0	106.8	0.989
Anammox bacteria *hzs*A gene	DNA	51.0	82.0	0.997	51.0	81.1	0.997	51.0	80.1	0.997
(*hzs*A_1597F/*hzs*A_1857R)	cDNA	51.0	80.0	0.998	51.0	80.2	0.997	51.0	80.4	0.996
AOB 16S rRNA gene	DNA	60.0	86.6	0.998	60.0	86.6	0.998	60.0	86.6	0.998
(CTO189F/CTO654R)	cDNA	60.0	84.0	0.998	60.0	84.0	0.998	60.0	84.0	0.998
AOB *amo*A gene	DNA	61.6	85.4	0.994	61.6	85.4	0.994	61.6	85.4	0.994
(AOB-amoAF/AOB-amoAR new)	cDNA	61.6	91.0	0.993	61.6	91.0	0.993	61.6	91.0	0.993

qPCR conditions: 95˚C 4 min; 40 × [95˚C 30 s, melting temperature (T_m) 40 s, 72˚C 30 s]; 80˚C 25 s

Table S3: Physical (CTD) and chemical (nutrient) properties of the bottom water (BW).

Parameter/ Sediment dbsf [cm]	Ammonium [µM]	Nitrite [µM]	Nitrate [µM]	Phosphate [µM]	Temperature [˚C]	Salinity [psu]	water depth [m]
**Feb-11**							
BW	3.0	0.6	6.8	1.0	5.0	34.4	43.0
**May-11**							
BW	1.9	0.1	2.0	0.7	8.6	34.3	44.0
**Aug-11**							
BW	1.6	0.1	0.5	0.5	15.4	34.7	45.0

Table S4: Total organic carbon (TOC) and total organic nitrogen (TON)

Parameter/ Sediment dbsf [cm]	TOC [%]	TON [%]
**Feb-11**		
0.5	0.22	0.02
1.5	0.22	bdl*
2.5	0.21	0.04
3.5	0.22	0.04
4.5	0.26	0.03
6.5	0.20	0.02
8.5	0.28	0.03
11.5	0.37	0.08
**May-11**		
0.5	0.20	0.02
1.5	0.30	0.05
2.5	0.25	0.04
3.5	0.27	0.03
4.5	0.38	0.08
6.5	0.25	0.05
8.5	0.15	0.03
11.5	0.21	0.02
**Aug-11**		
0.5	0.26	0.03
1.5	0.27	0.05
2.5	0.23	0.02
3.5	0.20	0.02
4.5	0.31	0.06
6.5	0.25	0.03
8.5	0.28	0.03
11.5	0.22	0.04

*bdl: below detection limit

Table S5: Spearman rank order correlation of all parameters observed in this study.

**Parameter**	**NH_4^+ [µM]**	**NO_3^- [µM]**	**PO_4^+ [µM]**	**TON [%]**	**AOA 16S rRNA gene (RNA:DNA ratio)**	**AOA *amo*A gene (DNA) [copies g^{-1}]**
Sediment depth [cm]	0.822***		0.801***			
NH_4^+ [µM]		0.734***				
NO_3^- [µM]						
PO_4^+ [µM]						
AOA 16S rRNA gene (DNA) [copies g^{-1}]						0.694***
AOA 16S rRNA gene (cDNA) [copies g^{-1}]					0.846***	
AOA *amo*A gene (DNA) [copies g^{-1}]						
AOA *amo*A gene (cDNA) [copies g^{-1}]						
AOA *amo*A gene (RNA:DNA ratio)						
Crenarchaeol IPLs total [area g^{-1}]						
HPH-crenarchaeol [area g^{-1}]						
TOC [%]				0.657***		
Anammox bacteria 16S rRNA gene (DNA) [copies g^{-1}]						
Anammox bacteria 16S rRNA gene (cDNA) [copies g^{-1}]						
Anammox bacteria 16S rRNA gene (RNA:DNA ratio)						
Anammox bacteria *hzs*A gene (DNA) [copies g^{-1}]						
Anammox bacteria *hzs*A gene (cDNA) [copies g^{-1}]						
Anammox *hzs*A gene (RNA:DNA ratio)						
PC Monoether ladderane [ng g^{-1}]						
AOB 16S rRNA gene (cDNA) [copies g^{-1}]						
AOB *amo*A (DNA) gene [copies g^{-1}]						
AOB *amo*A (cDNA) gene [copies g^{-1}]						
AOB *amo*A gene (RNA:DNA ratio)						

Correlation coefficients $r_s \geq 0.6$ and $r_s \leq -0.6$ with *P*-values ≤ 0.05 were reported. The degree of significance was indicated by using a single asterisk for *P*-values ≤ 0.05 and ≥ 0.01, two asterisks for *P*-values ≤ 0.01 and three asterisks for *P*-values ≤ 0.005.

Table S5: Spearman rank order correlation of all parameters observed in this study.

Parameter	AOA *amo*A gene (cDNA) [copies g^{-1}]	AOA *amo*A gene (RNA:DNA ratio)	Anammox 16S rRNA gene (DNA) [copies g^{-1}]	Anammox 16S rRNA gene (cDNA) [copies g^{-1}]
Sediment depth [cm]				
NH_4^+ [µM]				
NO_3^- [µM]			-0.647***	-0.743***
PO_4^+ [µM]	-0.745***	-0.739***		
AOA 16S rRNA gene (DNA) [copies g^{-1}]				
AOA 16S rRNA gene (cDNA) [copies g^{-1}]				
AOA *amo*A gene (DNA) [copies g^{-1}]			0.725***	
AOA *amo*A gene (cDNA) [copies g^{-1}]		0.994***		0.629***
AOA *amo*A gene (RNA:DNA ratio)				
Crenarchaeol IPLs total [area g^{-1}]				
HPH-crenarchaeol [area g^{-1}]				
TOC [%]				
Anammox bacteria 16S rRNA gene (DNA) [copies g^{-1}]				0.797***
Anammox bacteria 16S rRNA gene (cDNA) [copies g^{-1}]				
Anammox bacteria 16S rRNA gene (RNA:DNA ratio)		0.717***		
Anammox bacteria *hzs*A gene (DNA) [copies g^{-1}]				
Anammox bacteria *hzs*A gene (cDNA) [copies g^{-1}]				
Anammox *hzs*A gene (RNA:DNA ratio)				
PC Monoether ladderane [ng g^{-1}]				
AOB 16S rRNA gene (cDNA) [copies g^{-1}]				
AOB *amo*A (DNA) gene [copies g^{-1}]				
AOB *amo*A (cDNA) gene [copies g^{-1}]				
AOB *amo*A gene (RNA:DNA ratio)				

Correlation coefficients $r_s \geq 0.6$ and $r_s \leq -0.6$ with *P*-values ≤ 0.05 were reported. The degree of significance was indicated by using a single asterisk for *P*-values ≤ 0.05 and ≥ 0.01, two asterisks for *P*-values ≤ 0.01 and three asterisks for *P*-values ≤ 0.005.

Table S5: Spearman rank order correlation of all parameters observed in this study.

**Parameter**	**Anammox 16S rRNA (RNA:DNA ratio)**	**Anammox *hzs*A (DNA) [copies g^{-1}]**	**Anammox *hzs*A (cDNA) [copies g^{-1}]**	**Anammox *hzs*A (RNA:DNA ratio)**	**PC-ME ladderane [ng g^{-1}]**
Sediment depth [cm]					-0.662***
NH_4^+ [µM]					
NO_3^- [µM]					
PO_4^+ [µM]					
AOA 16S rRNA gene (DNA) [copies g^{-1}]					
AOA 16S rRNA gene (cDNA) [copies g^{-1}]					
AOA *amo*A gene (DNA) [copies g^{-1}]		0.662***			
AOA *amo*A gene (cDNA) [copies g^{-1}]	0.708***				
AOA *amo*A gene (RNA:DNA ratio)					
Crenarchaeol IPLs total [area g^{-1}]				0.797***	
HPH-crenarchaeol [area g^{-1}]				0.760***	
TOC [%]					
Anammox bacteria 16S rRNA gene (DNA) [copies g^{-1}]		0.848***		-0.751***	
Anammox bacteria 16S rRNA gene (cDNA) [copies g^{-1}]	0.748***	0.729***			
Anammox bacteria 16S rRNA gene (RNA:DNA ratio)					
Anammox bacteria *hzs*A gene (DNA) [copies g^{-1}]					
Anammox bacteria *hzs*A gene (cDNA) [copies g^{-1}]					
Anammox *hzs*A gene (RNA:DNA ratio)					
PC Monoether ladderane [ng g^{-1}]					
AOB 16S rRNA gene (cDNA) [copies g^{-1}]					
AOB *amo*A (DNA) gene [copies g^{-1}]					
AOB *amo*A (cDNA) gene [copies g^{-1}]			0.713***		
AOB *amo*A gene (RNA:DNA ratio)					

Correlation coefficients $r_s \geq 0.6$ and $r_s \leq -0.6$ with *P*-values ≤ 0.05 were reported. The degree of significance was indicated by using a single asterisk for *P*-values ≤ 0.05 and ≥ 0.01, two asterisks for *P*-values ≤ 0.01 and three asterisks for *P*-values ≤ 0.005.

Table S5: Spearman rank order correlation of all parameters observed in this study.

Parameter	AOB 16S rRNA (DNA) [copies g^{-1}]	AOB 16S rRNA (cDNA) [copies g^{-1}]	AOB 16S rRNA (RNA:DNA ratio)	AOB *amo*A (cDNA) [copies g^{-1}]	AOB *amo*A (RNA:DNA ratio)
Sediment depth [cm]					-0.646***
NH_4^+ [µM]					
NO_3^- [µM]					
PO_4^+ [µM]					
AOA 16S rRNA gene (DNA) [copies g^{-1}]					
AOA 16S rRNA gene (cDNA) [copies g^{-1}]					
AOA *amo*A gene (DNA) [copies g^{-1}]					
AOA *amo*A gene (cDNA) [copies g^{-1}]					
AOA *amo*A gene (RNA:DNA ratio)					
Crenarchaeol IPLs total [area g^{-1}]					
HPH-crenarchaeol [area g^{-1}]					
TOC [%]					
Anammox bacteria 16S rRNA gene (DNA) [copies g^{-1}]					
Anammox bacteria 16S rRNA gene (cDNA) [copies g^{-1}]					
Anammox bacteria 16S rRNA gene (RNA:DNA ratio)					
Anammox bacteria *hzs*A gene (DNA) [copies g^{-1}]					
Anammox bacteria *hzs*A gene (cDNA) [copies g^{-1}]		0.656***			
Anammox *hzs*A gene (RNA:DNA ratio)					
PC Monoether ladderane [ng g^{-1}]					
AOB 16S rRNA gene (cDNA) [copies g^{-1}]			0.620***		
AOB *amo*A (DNA) gene [copies g^{-1}]	0.617***				
AOB *amo*A (cDNA) gene [copies g^{-1}]		0.702***			
AOB *amo*A gene (RNA:DNA ratio)				0.887***	

Correlation coefficients $r_s \geq 0.6$ and $r_s \leq -0.6$ with *P*-values ≤ 0.05 were reported. The degree of significance was indicated by using a single asterisk for *P*-values ≤ 0.05 and ≥ 0.01, two asterisks for *P*-values ≤ 0.01 and three asterisks for *P*-values ≤ 0.005.

Figure S1

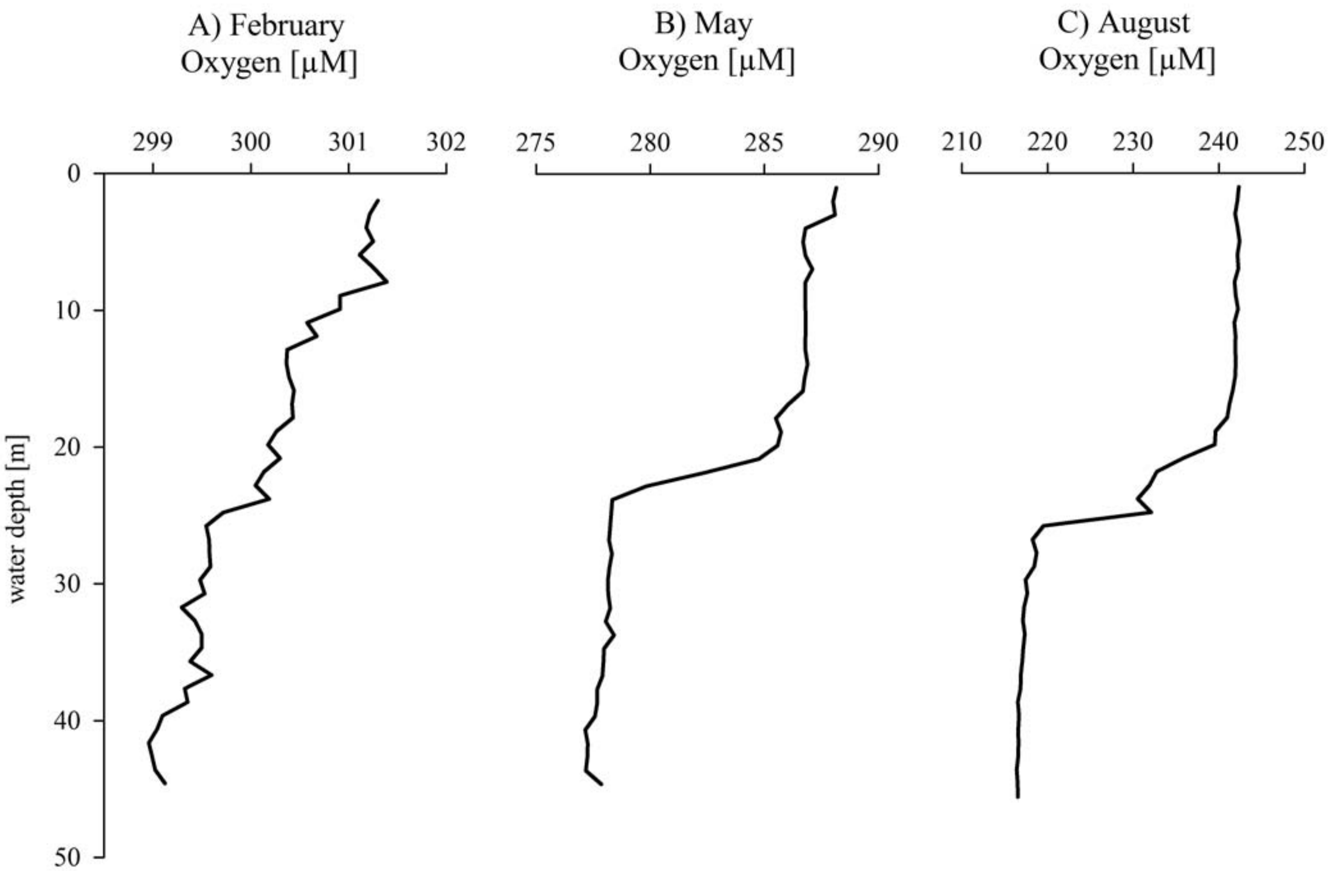

A) February
Oxygen [μM]
B) May
Oxygen [μM]
C) August
Oxygen [μM]
299
300
301
302
275
280
285
290
210
220
230
240
250
0
10
20
30
40
50
water depth [m]

Figure S2

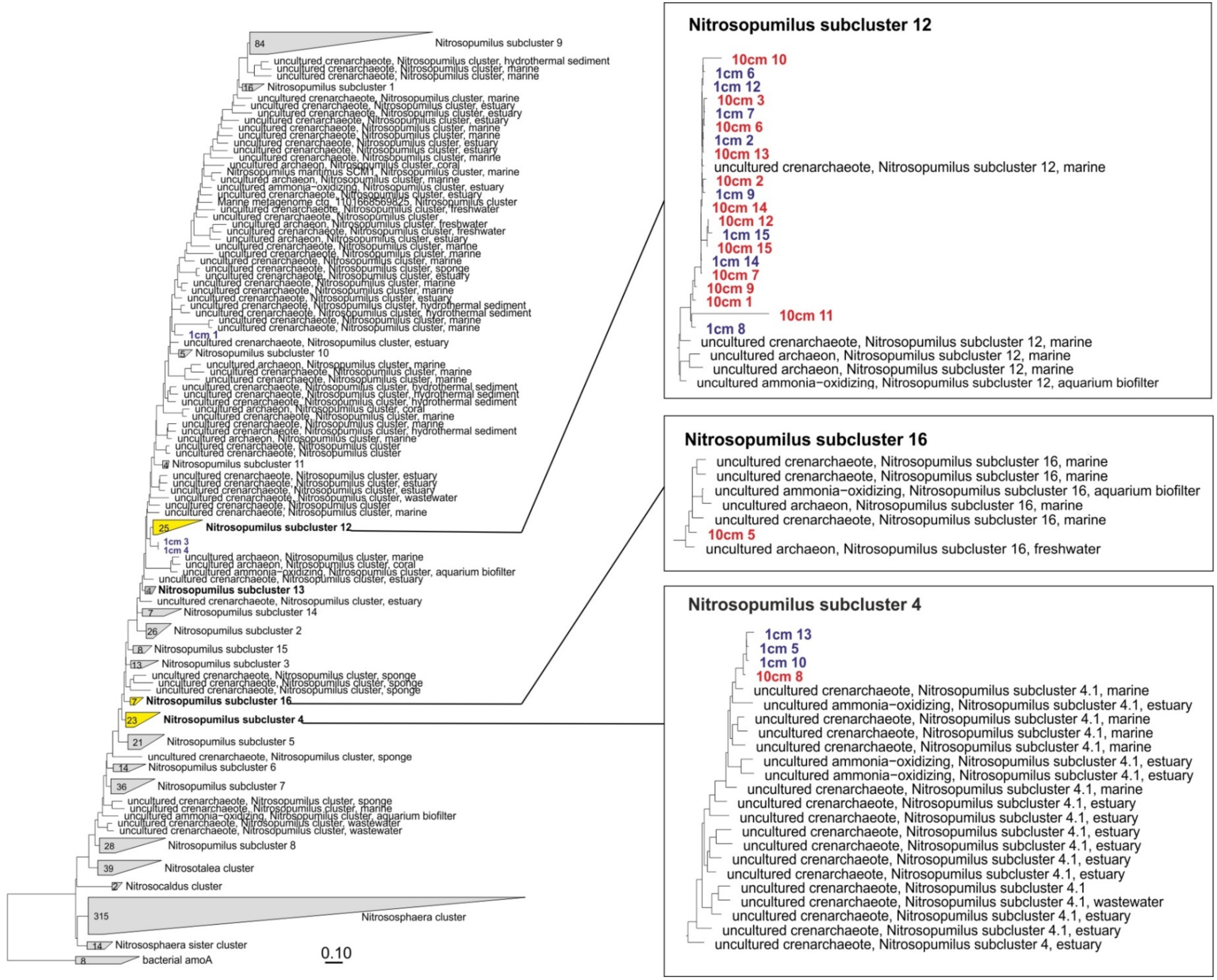

84 Nitrosopumilus subcluster 9
uncultured crenarchaeote, Nitrosopumilus cluster, hydrothermal sediment
16 Nitrosopumilus subcluster 1
1cm 1
uncultured crenarchaeote, Nitrosopumilus cluster, estuary
5 Nitrosopumilus subcluster 10
Nitrosopumilus subcluster 11
25 Nitrosopumilus subcluster 12
1cm 3
1cm 4
uncultured ammonia-oxidizing, Nitrosopumilus cluster, aquarium biofilter
4 Nitrosopumilus subcluster 13
uncultured crenarchaeote, Nitrosopumilus cluster, estuary
7 Nitrosopumilus subcluster 14
26 Nitrosopumilus subcluster 2
8 Nitrosopumilus subcluster 15
13 Nitrosopumilus subcluster 3
7 Nitrosopumilus subcluster 16
23 Nitrosopumilus subcluster 4
21 Nitrosopumilus subcluster 5
uncultured crenarchaeote, Nitrosopumilus cluster, sponge
14 Nitrosopumilus subcluster 6
36 Nitrosopumilus subcluster 7
uncultured ammonia-oxidizing, Nitrosopumilus cluster, aquarium biofilter
uncultured crenarchaeote, Nitrosopumilus cluster, wastewater
28 Nitrosopumilus subcluster 8
39 Nitrosotalea cluster
2 Nitrosocaldus cluster
315 Nitrososphaera cluster
14 Nitrososphaera sister cluster
8 bacterial amoA
0.10
Nitrosopumilus subcluster 12
10cm 10
1cm 6
1cm 12
10cm 3
1cm 7
10cm 6
1cm 2
10cm 13
uncultured crenarchaeote, Nitrosopumilus subcluster 12, marine
10cm 2
1cm 9
10cm 14
10cm 12
1cm 15
10cm 15
1cm 14
10cm 7
10cm 9
10cm 1
10cm 11
1cm 8
uncultured crenarchaeote, Nitrosopumilus subcluster 12, marine
uncultured archaeon, Nitrosopumilus subcluster 12, marine
uncultured archaeon, Nitrosopumilus subcluster 12, marine
uncultured ammonia-oxidizing, Nitrosopumilus subcluster 12, aquarium biofilter
Nitrosopumilus subcluster 16
uncultured crenarchaeote, Nitrosopumilus subcluster 16, marine
uncultured crenarchaeote, Nitrosopumilus subcluster 16, marine
uncultured ammonia-oxidizing, Nitrosopumilus subcluster 16, aquarium biofilter
uncultured archaeon, Nitrosopumilus subcluster 16, marine
uncultured crenarchaeote, Nitrosopumilus subcluster 16, marine
10cm 5
uncultured archaeon, Nitrosopumilus subcluster 16, freshwater
Nitrosopumilus subcluster 4
1cm 13
1cm 5
1cm 10
10cm 8
uncultured crenarchaeote, Nitrosopumilus subcluster 4.1, marine
uncultured ammonia-oxidizing, Nitrosopumilus subcluster 4.1, estuary
uncultured crenarchaeote, Nitrosopumilus subcluster 4.1, marine
uncultured crenarchaeote, Nitrosopumilus subcluster 4.1, marine
uncultured crenarchaeote, Nitrosopumilus subcluster 4.1, marine
uncultured ammonia-oxidizing, Nitrosopumilus subcluster 4.1, estuary
uncultured ammonia-oxidizing, Nitrosopumilus subcluster 4.1, estuary
uncultured crenarchaeote, Nitrosopumilus subcluster 4.1, marine
uncultured crenarchaeote, Nitrosopumilus subcluster 4.1, estuary
uncultured crenarchaeote, Nitrosopumilus subcluster 4.1, estuary
uncultured crenarchaeote, Nitrosopumilus subcluster 4.1, estuary
uncultured crenarchaeote, Nitrosopumilus subcluster 4.1, estuary
uncultured crenarchaeote, Nitrosopumilus subcluster 4.1, estuary
uncultured crenarchaeote, Nitrosopumilus subcluster 4.1, estuary
uncultured crenarchaeote, Nitrosopumilus subcluster 4.1
uncultured crenarchaeote, Nitrosopumilus subcluster 4.1, wastewater
uncultured crenarchaeote, Nitrosopumilus subcluster 4.1, estuary
uncultured crenarchaeote, Nitrosopumilus subcluster 4.1, estuary
uncultured crenarchaeote, Nitrosopumilus subcluster 4, estuary

Figure S3

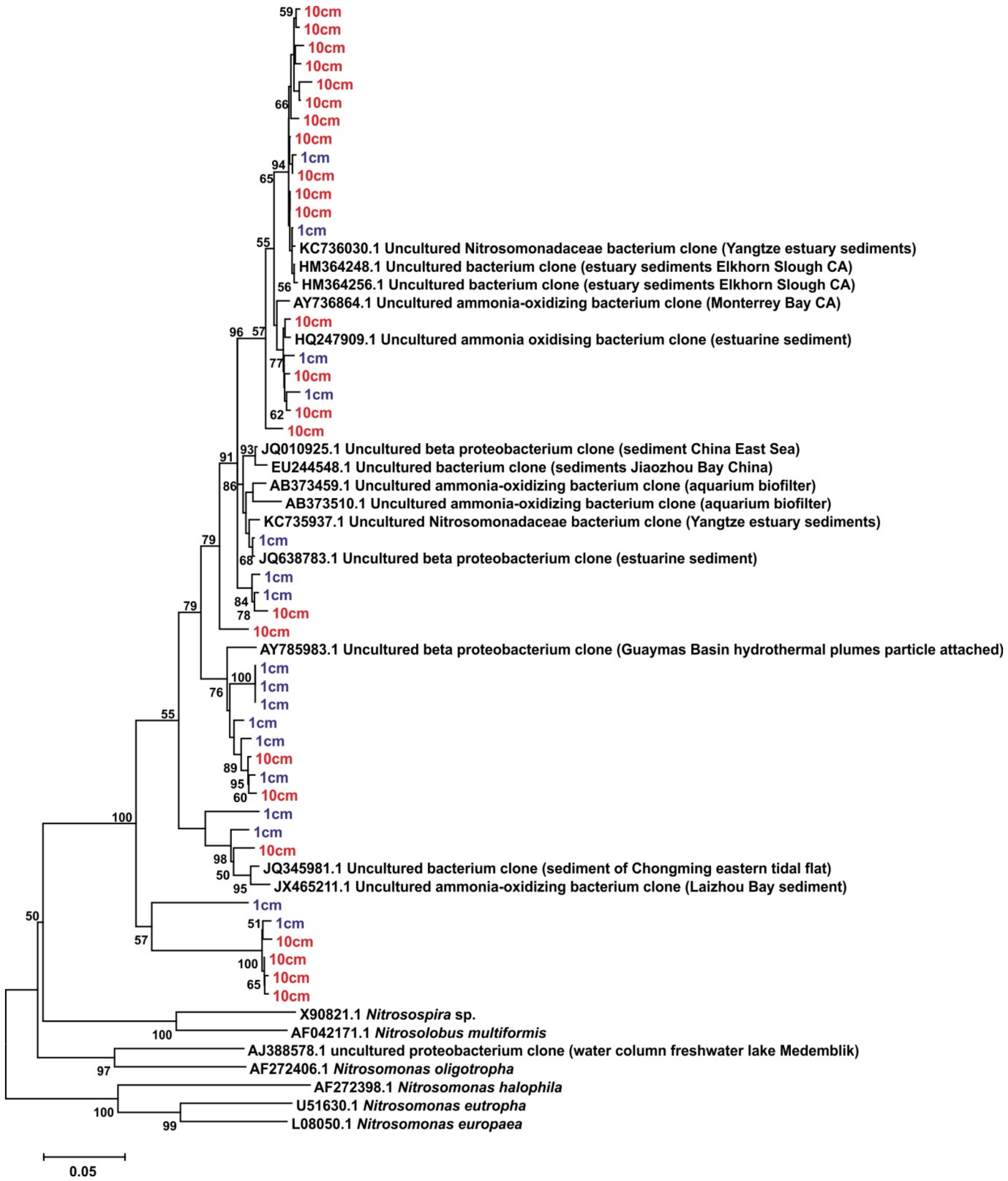

10cm
1cm
KC736030.1 Uncultured Nitrosomonadaceae bacterium clone (Yangtze estuary sediments)
HM364248.1 Uncultured bacterium clone (estuary sediments Elkhorn Slough CA)
HM364256.1 Uncultured bacterium clone (estuary sediments Elkhorn Slough CA)
AY736864.1 Uncultured ammonia-oxidizing bacterium clone (Monterey Bay CA)
HQ247909.1 Uncultured ammonia oxidising bacterium clone (estuarine sediment)
JQ010925.1 Uncultured beta proteobacterium clone (sediment China East Sea)
EU244548.1 Uncultured bacterium clone (sediments Jiaozhou Bay China)
AB373459.1 Uncultured ammonia-oxidizing bacterium clone (aquarium biofilter)
AB373510.1 Uncultured ammonia-oxidizing bacterium clone (aquarium biofilter)
KC735937.1 Uncultured Nitrosomonadaceae bacterium clone (Yangtze estuary sediments)
JQ638783.1 Uncultured beta proteobacterium clone (estuarine sediment)
AY785983.1 Uncultured beta proteobacterium clone (Guaymas Basin hydrothermal plumes particle attached)
JQ345981.1 Uncultured bacterium clone (sediment of Chongming eastern tidal flat)
JX465211.1 Uncultured ammonia-oxidizing bacterium clone (Laizhou Bay sediment)
X90821.1 *Nitrosospira* sp.
AF042171.1 *Nitrosolobus multiformis*
AJ388578.1 uncultured proteobacterium clone (water column freshwater lake Medemblik)
AF272406.1 *Nitrosomonas oligotropha*
AF272398.1 *Nitrosomonas halophila*
U51630.1 *Nitrosomonas eutropha*
L08050.1 *Nitrosomonas europaea*
0.05

Figure S4

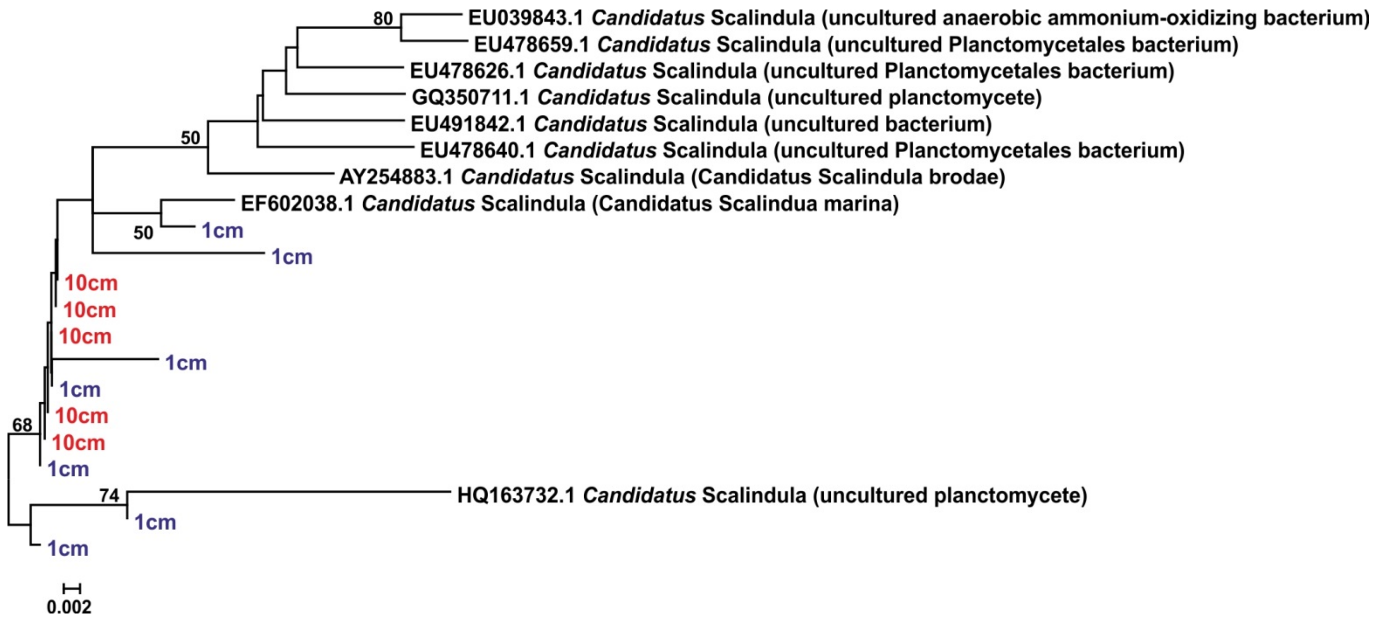
80
EU039843.1 *Candidatus* Scalindula (uncultured anaerobic ammonium-oxidizing bacterium)
EU478659.1 *Candidatus* Scalindula (uncultured Planctomycetales bacterium)
EU478626.1 *Candidatus* Scalindula (uncultured Planctomycetales bacterium)
GQ350711.1 *Candidatus* Scalindula (uncultured planctomycete)
EU491842.1 *Candidatus* Scalindula (uncultured bacterium)
50
EU478640.1 *Candidatus* Scalindula (uncultured Planctomycetales bacterium)
AY254883.1 *Candidatus* Scalindula (Candidatus Scalindula brodae)
EF602038.1 *Candidatus* Scalindula (Candidatus Scalindua marina)
50
1cm
1cm
10cm
10cm
10cm
1cm
1cm
10cm
68
10cm
1cm
74
HQ163732.1 *Candidatus* Scalindula (uncultured planctomycete)
1cm
1cm
0.002
